# Gut Virome Analysis of Cameroonians Reveals High Diversity of Enteric Viruses, Including Potential Interspecies Transmitted Viruses

**DOI:** 10.1128/mSphere.00585-18

**Published:** 2019-01-23

**Authors:** Claude Kwe Yinda, Emiel Vanhulle, Nádia Conceição-Neto, Leen Beller, Ward Deboutte, Chenyan Shi, Stephen Mbigha Ghogomu, Piet Maes, Marc Van Ranst, Jelle Matthijnssens

**Affiliations:** a Department of Microbiology and Immunology, Rega Institute for Medical Research, Laboratory of Viral Metagenomics, KU Leuven-University of Leuven, Leuven, Belgium; b Department of Microbiology and Immunology, Rega Institute for Medical Research, Laboratory for Clinical and Epidemiological Virology, KU Leuven-University of Leuven, Leuven, Belgium; c Department of Biochemistry and Molecular Biology, Biotechnology Unit, Molecular and Cell Biology Laboratory, University of Buea, Buea, Cameroon; Pennsylvania State University

**Keywords:** Cameroon, gut, human, virome

## Abstract

Diarrhea remains one of the most common causes of deaths in children. A limited number of studies have investigated the prevalence of enteric pathogens in Cameroon, and as in many other African countries, the cause of many diarrheal episodes remains unexplained. A proportion of these unknown cases of diarrhea are likely caused by yet-unidentified viral agents, some of which could be the result of (recent) interspecies transmission from animal reservoirs, like bats. Using viral metagenomics, we screened fecal samples of 221 humans (almost all with gastroenteritis symptoms) between 0 and 89 years of age with different degrees of bat contact. We identified viruses belonging to families that are known to cause gastroenteritis such as *Adenoviridae*, *Astroviridae*, *Caliciviridae*, *Picornaviridae*, and *Reoviridae*. Interestingly, a mammalian orthoreovirus, picobirnaviruses, a smacovirus, and a pecovirus were also found. Although there was no evidence of interspecies transmission of the most common human gastroenteritis-related viruses (*Astroviridae*, *Caliciviridae*, and *Reoviridae*), the phylogenies of the identified orthoreovirus, picobirnavirus, and smacovirus indicate a genetic relatedness of these viruses identified in stools of humans and those of bats and/or other animals. These findings points out the possibility of interspecies transmission or simply a shared host of these viruses (bacterial, fungal, parasitic, …) present in both animals (bats) and humans. Further screening of bat viruses in humans or vice versa will elucidate the epidemiological potential threats of animal viruses to human health. Furthermore, this study showed a huge diversity of highly divergent novel phages, thereby expanding the existing phageome considerably.

**IMPORTANCE** Despite the availability of diagnostic tools for different enteric viral pathogens, a large fraction of human cases of gastroenteritis remains unexplained. This could be due to pathogens not tested for or novel divergent viruses of potential animal origin. Fecal virome analyses of Cameroonians showed a very diverse group of viruses, some of which are genetically related to those identified in animals. This is the first attempt to describe the gut virome of humans from Cameroon. Therefore, the data represent a baseline for future studies on enteric viral pathogens in this area and contribute to our knowledge of the world’s virome. The studies also highlight the fact that more viruses may be associated with diarrhea than the typical known ones. Hence, it provides meaningful epidemiological information on diarrhea-related viruses in this area.

## Abstract (precis)

Despite the availability of diagnostic tools for different enteric viral pathogens, a large fraction of human cases of gastroenteritis remains unexplained. This could be due to pathogens not tested for or novel divergent viruses of potential animal origin. Fecal virome analyses of Cameroonians showed a very diverse group of viruses, some of which are genetically related to those identified in animals. This is the first attempt to describe the gut virome of humans from Cameroon. Therefore, the data represent a baseline for future studies on enteric viral pathogens in this area and contribute to our knowledge of the world’s virome. The studies also highlight the fact that more viruses may be associated with diarrhea than the typical known ones. Hence, it provides meaningful epidemiological information on diarrhea-related viruses in this area.

## INTRODUCTION

Diarrhea is the second most common cause of death worldwide and accounts for about 8 to 9% of the 5.9 million yearly deaths in children under the age of 5 (1, 2). Most of these deaths occur in Southeast Asia and sub-Saharan Africa (3, 4). The chances of infection with enteric viruses are higher in developing countries than developed countries, probably due to suboptimal sanitation and hygienic conditions and low quality of drinking water, especially in rural areas (5). In Cameroon, a limited number of studies have investigated the prevalence of enteric pathogens as the cause of gastroenteritis in humans. These studies mainly focused on the epidemiology of a limited number of pathogens such as rotavirus, norovirus, and enteroviruses, revealing significant differences in the prevalence of these viruses in different settings and time periods (4, 6, 7). In parts of Cameroon, a high prevalence of several enteric viruses such as enterovirus, norovirus, rotavirus, and adenovirus was found in children and adults (8). Generally in Africa, many episodes of gastroenteritis remain unexplained as no etiological agent is determined (9, 10). A proportion of the unexplained gastroenteritis cases are likely due to other known viruses, for which no tests were performed. However, a part of these gastroenteritis cases could also be caused by novel viral agents.

Transmission of these enteric viruses is predominantly fecal-oral, and humans are constantly exposed to these viruses through various routes (11). One of these routes is zoonosis from reservoirs in wild or domestic animals, either by insect vectors or by exposure to animal droppings or tissues. One rich but, until recently, underappreciated reservoir of emergent viruses is bats. Of the ∼5,500 known terrestrial species of mammals, about 20% are bats (12). Several viruses pathogenic to humans are believed to have originated in bats over the last several years, including severe acute respiratory syndrome (SARS)- and Middle East respiratory syndrome (MERS)-related coronaviruses, as well as filoviruses, such as Ebola and Marburg viruses, or henipaviruses, such as Nipah and Hendra viruses (13–18).

In the Southwest region of Cameroon, bats are hunted and eaten. Such close interactions provide ample opportunity for zoonotic events to occur (19).

Previously, we identified a plethora of known and novel eukaryotic viruses in Cameroonian fruit bats using a viral metagenomics approach, including viruses known to cause gastroenteritis in humans (sapovirus, sapelovirus, and rotaviruses A and H) and those not yet associated with gastroenteritis (bastrovirus and picobirna-like viruses) (20–23). In the current study, we metagenomically screened 221 human fecal samples collected in the same region (where bats are hunted and eaten), to assess (i) if any viruses of animal origin could be identified and (ii) which known human gastrointestinal viruses were present. These fecal samples were collected from children less than a year old to adults of more than 60 years who had gastroenteritis and/or were in contact with bats. Additionally, since the gut virome typically contains both eukaryotic and prokaryotic viruses (phages), of which the latter usually represents the largest fraction of the gut virome, we also analyzed the phageome of these samples.

## RESULTS

### Sample characterization.

A total of 221 human fecal samples (131 from Kumba and 90 from Lysoka) were collected from two hospitals in the Southwest region of Cameroon, for viral metagenomics screening. From these fecal samples, a total of 63 pools were constituted in categories based on age, bat contact status, and location (see Table S1 in the supplemental material). Illumina sequencing of all the 63 human pools generated in total approximately 708 million raw paired-end (PE) reads (between 4.3 and 53.4 million reads per pool). After trimming, 67% of the reads (471 million) were retained and 86% of these retained trimmed reads (405 million) were annotated using Diamond. Of these, 18% (74 million) could be attributed as viral.

10.1128/mSphere.00585-18.8 TABLE S1 Metadata pools and reads. Download Table S1, PDF file, 0.1 MB. Copyright © 2019 Yinda et al. 2019 Yinda et al. This content is distributed under the terms of the Creative Commons Attribution 4.0 International license.

### NGS viral read distribution/abundances.

In each of the categories of pools, phages make up at least 84% of the total number of viral reads while the maximum proportion of eukaryotic viral reads is 16%. A similar annotation profile was observed for pools of patients in different age groups, different locations, and different bat contact statuses (Fig. S1).

10.1128/mSphere.00585-18.1 FIG S1 Proportion of reads belonging to viral groups stratified based on different parameters. Dark green bars indicate the proportion of reads annotated as bacteriophages and bars in light green indicate the proportion of eukaryotic virus reads. (A) Proportion of viral reads in different age categories. (B) Proportion of viral reads in different contact statuses. (C) Proportion of viral reads in different locations. Download FIG S1, PDF file, 0.2 MB. Copyright © 2019 Yinda et al. 2019 Yinda et al. This content is distributed under the terms of the Creative Commons Attribution 4.0 International license.

**FIG 1:**
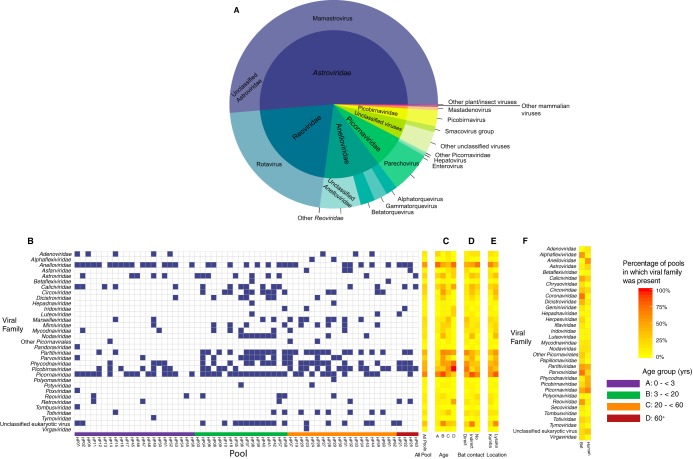
(A) Overview of the most abundant viral families and genera identified in humans in this study based on assigned reads. Low-abundance mammalian viruses not present in this figure belong to the *Caliciviridae*, *Circoviridae*, *Geminiviridae*, *Hepadnaviridae*, *Nodaviridae*, *Parvoviridae*, and unclassified *Picornavirales*. Other low-abundance plant/insect viruses not in this figure are *Alphatetraviridae*, *Betatetraviridae*, *Luteoviridae*, *Maiseilleviridae*, *Partitiviridae*, *Peribunyaviridae*, *Phycodnaviridae*, *Pithoviridae*, *Totiviridae*, and *Tymoviridae.* The viruses of families that could not be assigned to any known genus are referred to as unclassified viruses. Families represented by fewer than 100 reads were excluded. (B to E) Heat map of the presence of eukaryotic viral families in feces from all 63 pools in relation to different parameters (B, individual pools; C, age; D, bat contact status; E, location). Color code for panel B: blue square, presence of viral family in pool (more than 0.001% of total reads of that pool); white square, absence of viral family in pool (less than 0.001% of total reads of that pool). (F) Heat map of viral family presence in human and bat pools.

Further analysis of eukaryotic viral reads revealed that at least 70% of the reads mapped to viruses of the families *Astroviridae*, *Reoviridae*, and *Anelloviridae* (Fig. 1A). Other viruses were also present, particularly those that are known to cause gastroenteritis belonging to the families *Adenoviridae*, *Caliciviridae* (*Sapovirus* and *Norovirus*), and *Picornaviridae* (of which about 60% were enteroviruses [Fig. 1B and Fig. S2]). Also, reads from viruses known to cause other human diseases (*Parvoviridae*) or other animal diseases (*Circoviridae*) or not associated with any diseases at all (*Picobirnaviridae*) were present in variable numbers in the different groups (Fig. 1B to E). The rest of the viral families were either plant- or insect-associated viruses. Notably, in age groups A to D, the percentage of pools in which *Picobirnaviridae* viruses were present increased with age with low percentages in age groups A and B (Fig. 1C). Also, the percentages of pools positive for anelloviruses differed with respect to age, with higher percentages in young children and the elderly. Further, there were no observable trends in the percentage of eukaryotic viral presence with respect to bat contact status or location (Fig. 1D and E).

10.1128/mSphere.00585-18.2 FIG S2 Heat map of the presence of eukaryotic viral genera in feces from all 63 pools grouped according to age. Download FIG S2, PDF file, 0.2 MB. Copyright © 2019 Yinda et al. 2019 Yinda et al. This content is distributed under the terms of the Creative Commons Attribution 4.0 International license.

Figure 1F shows a heat map of the percentage of pools in which eukaryotic viral families were present in human and bat pools, while Fig. S3 compares the viral presence in human and bats at the genus level (23). *Astroviridae* (*Mamastrovirus*), *Calciviridae* (*Sapovirus*), *Picornaviridae* (*Parechovirus*), and *Reoviridae* (*Rotavirus*), viral families known to cause gastroenteritis in humans, were identified in both bat and human pools from the same region. Also, mammalian viruses not yet established to cause gastroenteritis (*Picobirnaviridae*, *Circoviridae*, and *Parvoviridae* [*Bocaparvovirus*]) were also common in both bats and humans from the same regions (Fig. 1F and Fig. S3).

10.1128/mSphere.00585-18.3 FIG S3 Heat map of the presence of eukaryotic viral genera in human and bat pools from the same region. Download FIG S3, PDF file, 0.3 MB. Copyright © 2019 Yinda et al. 2019 Yinda et al. This content is distributed under the terms of the Creative Commons Attribution 4.0 International license.

### Phylogeny of eukaryotic viruses.

In this study, we focused on viruses from which near- complete genomes were obtained, particularly those that are known to cause viral gastroenteritis (belonging to the *Astroviridae*, *Caliciviridae* [norovirus and sapovirus], *Picornaviridae* [enterovirus, parechovirus, cosavirus], *Parvoviridae*, *Reoviridae*, and *Adenoviridae* [human mastadenovirus]). Furthermore, we also looked at other viruses not fully proven to cause gastroenteritis in humans but which have only sporadically been associated with gastroenteritis, like *Picobirnaviridae* and small circular single-stranded DNA viruses.

Phylogenetic analysis was done for each of the selected viruses using the protein or nucleotide sequences of suitable conserved regions and representative members of their viral family, genus, or species.

### Reoviridae.

*Reoviridae* is a large viral family of segmented dsRNA viruses with a wide host range. They are further divided into two subfamilies and 15 genera. Genomes of viruses belonging to the *Reoviridae* contain 9 to 12 segments (24). In total, *Reoviridae* reads were found in 6 pools, and (nearly) complete genomes of 2 viruses of the family *Reoviridae* were obtained from pool HP55. Samples in this pool were from two diarrheic children (less than 5 years), originating from Kumba and without contact with bats.

### Mammalian orthoreovirus.

**FIG 2:**
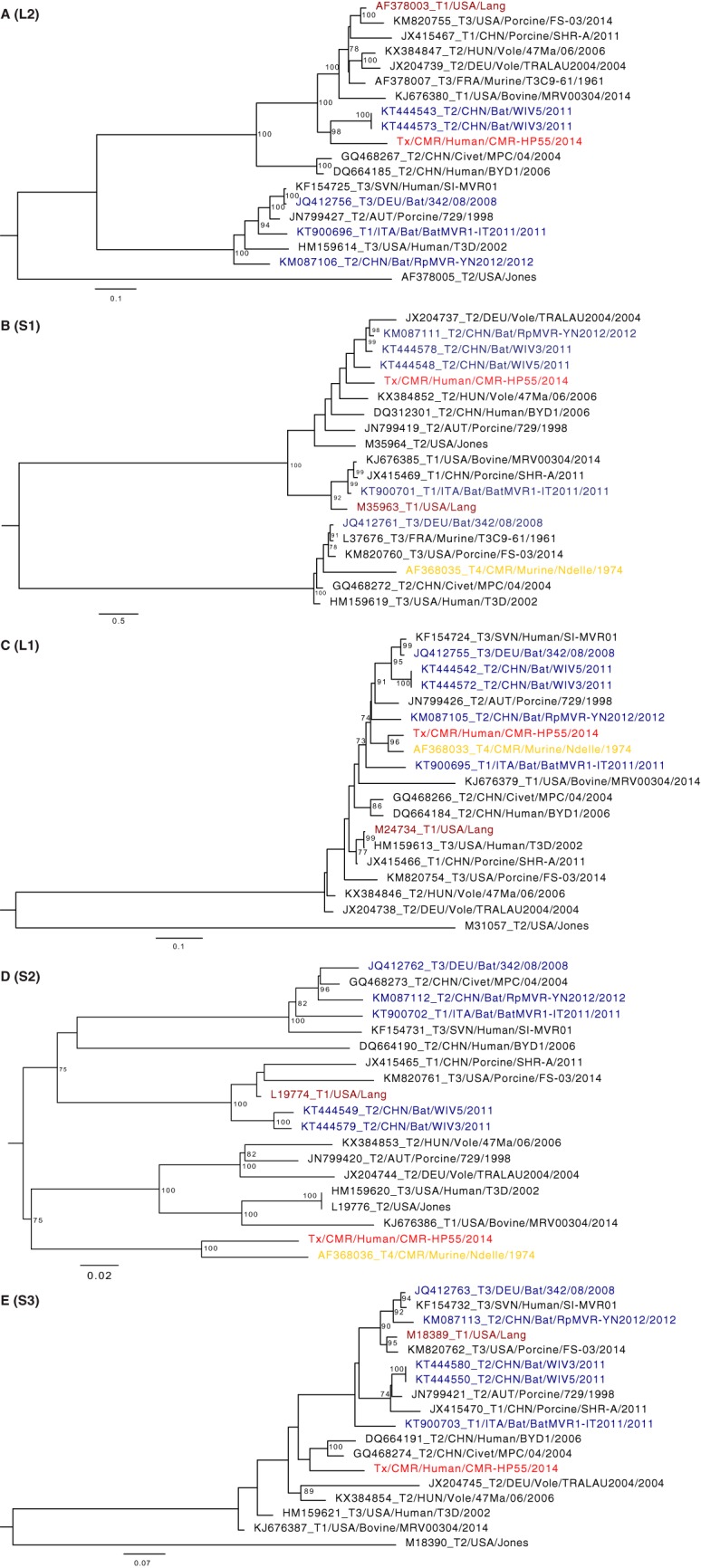
Maximum likelihood phylogenetic trees based on the nucleotide sequences of the L2, S1, L1, S2, and S3 coding segments of the novel MORV (indicated in red) and representative strains from GenBank showing 3 patterns of clustering with respect to the novel strain: A and B, clustering of novel strain with bat strains from China; C and D, clustering of novel strain with murine strain from Cameroon; and E, clustering of novel strain with human and civet strains from China. Trees were constructed using the GTR+G+I nucleotide substitution model using RAxML, with the autoMRE flag, which enables *a posteriori* bootstrapping analysis. Only bootstrap values greater than 70% are shown. Bars indicate nucleotide substitutions per site.

Mammalian orthoreoviruses (MORVs) contain 10 segments, L1 to L3, M1 to M3, and S1 to S4, coding for 12 to 13 proteins (24, 25). A MORV strain was identified represented by 16,913 reads (0.4% of all viral reads of the pool). Phylogenetic analysis based on the nucleotide sequences of each of the 10 segments of this MORV (Fig. 2 and Fig. S4) showed topological incongruence with four distinctive patterns. Based on segments L2 and S1, this strain clustered with bat strains WIV3 and WIV5 from China with 86% and 70% nucleotide (nt) identity, respectively (Fig. 2A and B). For the L1 and S2 segments, the human strain clustered with the Ndelle murine strain, also from Cameroon, with 95% and 92% nt identity, respectively (Fig. 2C and D). On the other hand, segment S3 of the Cameroonian MORV strain clustered with a human strain and a civet MORV strain from China (88% and 89% nt identity, respectively [Fig. 2E]). The rest of the segments (L3, M1 to M3, and S4) did not cluster together clearly with any of the abovementioned strains (Fig. S4).

10.1128/mSphere.00585-18.4 FIG S4 Maximum likelihood phylogenetic trees based on the nucleotide sequences of the L3, M1 to M3, and S4 coding segments of the novel MORV (indicated in red) and representative strains from GenBank. Trees were constructed using the GTR+G+I nucleotide substitution model using RAxML, with the autoMRE flag, which enables *a posteriori* bootstrapping analysis. Only bootstrap values greater than 70% are shown. Bars indicate nucleotide substitutions per site. Download FIG S4, PDF file, 0.2 MB. Copyright © 2019 Yinda et al. 2019 Yinda et al. This content is distributed under the terms of the Creative Commons Attribution 4.0 International license.

### Rotavirus A.

**FIG 3:**
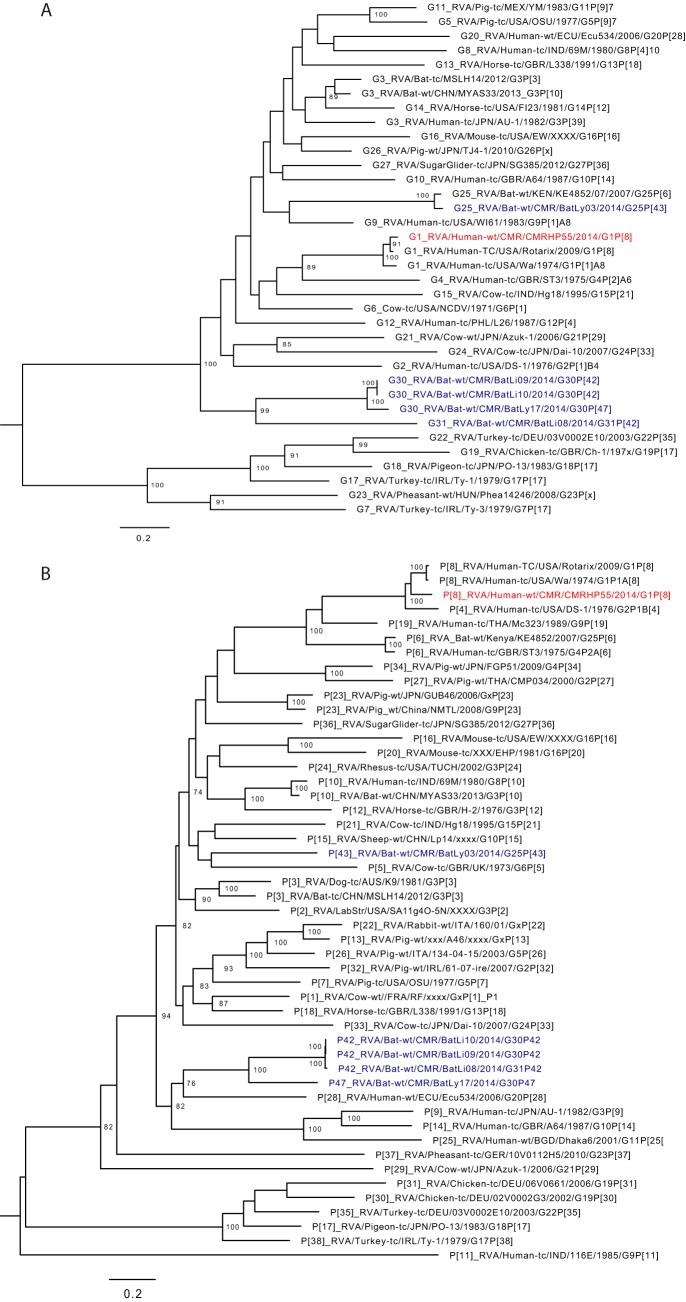
Phylogenetic trees of full-length ORF nucleotide sequences of RVA VP4 (A) and VP7 (B) gene segments showing close genetic relatedness to typical Wa-like genotype G1P[8] strains. Red, Cameroonian human RVA strain identified in this study; blue, Cameroonian bat RVA strains. Trees were constructed using the GTR+G+I nucleotide substitution model using RAxML, with the autoMRE flag, which enables *a posteriori* bootstrapping analysis. Only bootstrap values greater than 70% are shown. Bars indicate nucleotide substitutions per site.

Rotavirus A (RVA) contains 11 segments coding for 11 or 12 proteins: VP1 to VP4, VP6, VP7, and NSP1 to NSP6 (26, 27). We identified a near-complete RVA sequence which made up 99% (4.3 million) of the eukaryotic viral reads of that pool. The NSP3 segment was not identified in the sample. The VP7 gene of this strain was genetically most related to RVA/Human-tc/USA/Wa/1974/G1P1A[8] and RVA/Human-TC/USA/Rotarix/2009/G1P[8] (nt identity of 92 and 97%, respectively) while the VP4 gene was 90% identical to the same strains. The phylogenetic trees of the remaining segments shared the same clustering pattern (Fig. 3A and B and Fig. S5). According to the rotavirus classification scheme, this strain is a typical Wa-like G1P[8] named RVA/Human-wt/CMR/CMRHP55/2014/G1P[8]. CMRHP55 was distantly related to bat RVA strains identified from the same regions (only 69 to 71% nt identity).

10.1128/mSphere.00585-18.5 FIG S5 Phylogenetic trees of full-length ORF nucleotide sequences of RVA VP1 to VP3, VP6, NSP1, NSP2, NSP4, and NSP5 gene segments showing close genetic relatedness to typical Wa-like genotype G1P[8] strains. Red, Cameroonian human RVA strain identified in this study; blue, Cameroonian bat RVA strains. Trees were constructed using the GTR+G+I nucleotide substitution model using RAxML, with the autoMRE flag, which enables *a posteriori* bootstrapping analysis. Only bootstrap values greater than 70% are shown. Bars indicate nucleotide substitutions per site. Download FIG S5, PDF file, 0.3 MB. Copyright © 2019 Yinda et al. 2019 Yinda et al. This content is distributed under the terms of the Creative Commons Attribution 4.0 International license.

### Picornaviridae.

The *Picornaviridae* represent a large family of small, cytoplasmic, nonenveloped icosahedral ssRNA viruses consisting of 80 species, grouped into 35 genera. They have a genome of 7.1 to 8.9 kb in size and are most often composed of a single ORF encoding a polyprotein flanked by a 5′ and 3′ UTR (28). The members of the family *Picornaviridae* can cause gastroenteritis, meningitis, encephalitis, paralysis (nonpolio and polio-type), myocarditis, hepatitis, upper respiratory tract infections, and diabetes (29, 30). Out of the 63 pools, 41 contained *Picornaviridae* reads, making the *Picornaviridae* the eukaryotic viral family of which reads could be identified in the highest number of pools.

### Enterovirus.

**FIG 4:**
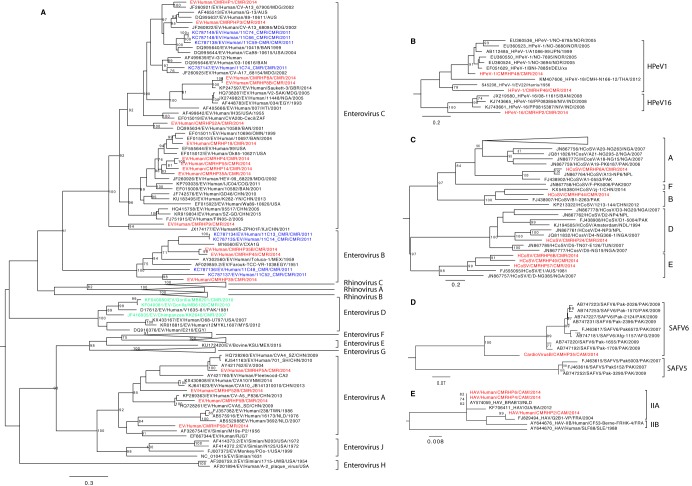
Phylogenetic relationships of picornaviruses identified in this study: A, genus *Enterovirus*; B, genus *Parechovirus*; C, genus *Cosavirus*; D, genus *Cardiovirus*; E, species *Hepatovirus A*. Phylogenetic trees were based on the nucleotide sequences of the VP1-P2A region for the species *Hepatovirus A* and the VP1 region for the rest of the genera. All the trees were constructed using the GTR+G+I nucleotide substitution model using RAxML, with the autoMRE flag, which enables *a posteriori* bootstrapping analysis. Only bootstrap values greater than 70% are shown. Bars indicate nucleotide substitutions per site. Red, novel strains from this study; blue, human Cameroonian enterovirus strains from other studies; green, animal enterovirus strains from Cameroon.

The genus *Enterovirus* (EV) consists of 15 species: *Enterovirus A* to *L* and *Rhinovirus A* to *C*. EV A, B, C, and D are found in humans; E and F in cattle; G in pigs; H, J, and L in monkeys; K in rodents; and species I in dromedary camels (http://www.picornaviridae.com). In this study, eighteen (nearly) complete genomes of EVs were obtained. The strains were named EV/Human/CMRHPxx/CMR/2014, here referred to as EV-CMRHPxx. All eighteen genomes were found in pools of age groups A and B (<3 and 3 to 20 years, respectively). Eight of these were identified in age group A, three (EV-CMRHP1, 5A, and 5B) of which were pools consisting of samples of infants who had indirect contact with bats while the rest (EV-CMRHP14, 45, 52A, 52B, and 55) were those that had no contact with bats. The ten other strains were identified in pools belonging to age group B, three of which had direct contact with bats (EV-CMRHP8A, 8B, and 9), 5 indirect contact (EV-CMRHP3, 4, 35A, 35B, and 39) and two with no contact (EV-CMRHP18 and 58). Based on the phylogenetic analysis of the VP1 nucleotide sequences, the EVs found in this study were quite divergent from each other, belonging to three different species of *Enterovirus*, *A*, *B*, and *C* (Fig. 4A). Most of the strains belonged to the *Enterovirus C* clade (EV-CMRHP1, 3, 4, 8A, 8B, 9, 14, 18, 35A, 52A, and 55), while EV-CMRHP35B, 39, and 45 clustered within the *Enterovirus B* genotype, and EVCMRHP5A, 5B, 52B, and 58 in the genogroup *Enterovirus A*. Some pools had multiple strains of EV present, and some of these clustered together (CMRHP8A and 8B: vaccine type PV-3), whereas other pools contained distinct EV species (EV-CMRHP35A and 35B; 52A and 52B). The presence of vaccine strains (PV-3) in pool HP8 probably indicates recent vaccination events of the infants in this pool. Apart from EV-CMRHP39 (which clustered with 11C52_CMR), all the EV strains identified here were distantly related to those previously identified in the Far North region of Cameroon (31). Furthermore, none of the human strains from Cameroon were related to any of the animal EV strains (from chimp or gorilla). A summary of the detailed classification of these EVs using an online typing tool (32) is shown in Table 1.

**TABLE 1:** Classification of Cameroonian EVs^*a*^

Strain name^*b*^	RefSeq	Enterovirus species	% nt identity	Type	% type support
EV-CMRHP1	NC_002058	Enterovirus C	76.9	CV-A13	100.0
EV-CMRPHP3	NC_002058	Enterovirus C	80.1	CV-A13	100.0
EV-CMRHP5A	NC_001612	Enterovirus A	84.7	CV-A4	100.0
EV-CMRHP5B	NC_001612	Enterovirus A	83.1	CV-A5	100.0
EV-CMRHP8A	NC_002058	Enterovirus C	99.9	PV-3	100.0
EV-CMRHP8B	NC_002058	Enterovirus C	99.9	PV-3	100.0
EV-CMRHP9	NC_002058	Enterovirus C	76.8	EV-C116	100.0
EV-CMRHP14	NC_002058	Enterovirus C	79.6	EV-C99	99.0
EV-CMRHP4	NC_002058	Enterovirus C	79.5	EV-C99	100.0
EV-CMRHP18	NC_002058	Enterovirus C	83.1	EV-C99	100.0
EV-CMRHP35A	NC_002058	Enterovirus C	79.8	EV-C99	100.0
EV-CMRHP35B	NC_001472	Enterovirus B	81.4	E-20	100.0
EV-CMRHP45	NC_001472	Enterovirus B	80.9	E-20	100.0
EV-CMRHP52A	NC_002058	Enterovirus C	81.6	CV-A11	100.0
EV-CMRHP52B	NC_001612	Enterovirus A	77.3	CV-A3	100.0
EV-CMRHP55	NC_002058	Enterovirus C	80.0	EV-C99	100.0
EV-CMRHP58	NC_001612	Enterovirus A	76.0	EV-A90	96.0
EV-CMRHP39	NC_001472	Enterovirus B	83.5	EV-B88	100.0

a The classification was done with an online typing tool (32).

b Full name of enterovirus strain EV/Human/CMRHPxx/CMR/2014.

### Parechovirus.

The genus *Parechovirus* is comprised of two species, *Parechovirus A* (human parechovirus [HPeV]) and *Parechovirus B* (Ljungan virus, isolated from bank voles) (33). HPeV is subdivided into 19 types (HPeV1 to -19). HPeV is associated with mild gastrointestinal or respiratory illness; however, severe disease conditions, such as meningitis/encephalitis, acute flaccid paralysis, and neonatal sepsis, may occur (34–36). Here, three (nearly) complete HPeVs were identified in pools HP2, HP46, and HP48 with sequence lengths of 7,142 bp, 7,202 bp, and 7,219 bp, respectively, collected from children less than 3 years old (age group A). In terms of bat contact status, they were in pools of those either in indirect contact with bats (HP2 and HP48) or without contact (HP46). They were all distantly related to each other, with HPeV-CMRHP46 and HPeV-CMRHP48 having the highest identity (76% and 86% nt and aa identity, respectively). Phylogenetically, HPeVs in HP46 and in HP48 fell into a clade of type 1 HPeVs (Fig. 4B). The HPeV in HP46 clustered together with HPeV1/Harris strain with 76% nt identity, while CMRHP48 clustered closely with Japanese and Norwegian strains A1086-99 and NO-3694 (84 to 90% nt identity). Furthermore, HPeV-CMRHP2 clustered distantly with type 16 HPeVs from China and Bangladesh with only 70 to 71% nt identity. Considering the 75% identity demarcation for HPeV types (37, 38), this strain potentially represents a novel type.

### Cosavirus.

The genus *Cosavirus* consists of five species (*Cosavirus A*, *B*, and *D* to *F*), which have been associated with gastroenteritis in children (39). Six near-complete human cosavirus (HCoSV) genomes were identified: 1 from children less than 3 years old (HP49), 3 from those between 3 and <20 years old (HP6A and HP6B, HP57), and 2 from pools of individuals between 20 and <60 years old (HP44, HP24). Some of these pools had direct or indirect contact with bats (HP6, HP24, and HP44), while others had no contact with bats (HP49 and HP57). Phylogenetic analysis (Fig. 4C) showed that cosaviruses from HP6B, HP49, and HP57 formed a clade with two other strains from Australia and Nigeria (HCoSV/E1/AUS and HCoSV/NG385/NGA) in species HCoSV E. Meanwhile the strains in HP6A, HP24, and HP44 clustered with HCoSV in species A, D, and B, respectively. Therefore, it seems that humans in Cameroon host a diverse range of cosaviruses.

### Cardiovirus.

The genus *Cardiovirus* consists of three species, *Cardiovirus A* to *C*. Species B includes Saffold virus (SafV) infecting humans. It has been found in cases with acute flaccid paralysis, respiratory tract infections, and diarrhea in China (40–42). Here, we found a near-complete genome of a SafV in one pool (HP35) belonging to the age group between 3 and <20 years old who had indirect contact with bats. The VP1 segment of the identified SafV was 72 to 74% and 78 to 80% identical (on nt level) to SafV strains in types 5 and 6, respectively. Phylogenetic analysis based on the VP1 region confirmed the clustering of the novel strain between types 5 and 6 with more phylogenetic relatedness to type 6 (Fig. 4D). Hence, this novel SafV strain may be a distant member of type 6 or represent a new type.

### Hepatovirus A.

Hepatitis A virus (HAV), now *Hepatovirus A*, belongs to the genus *Hepatovirus*, which consists of nine species (*Hepatovirus A* to *I*). The *Hepatovirus A* species is comprised of a single serotype, HAV, subdivided into human and simian viruses (43). It causes acute hepatitis throughout the world (44). There were three (nearly) complete HAV genomes in pools HP2, HP4, and HP6, all of which were pools from those in direct (HP6) or indirect (HP2 and HP4) contact with bats. These strains were either from infants less than 3 years old (HP2) or from children between 3 and <20 years old (HP4 and HP6). Based on the VP1-P2A region, the nt identity between these strains was 98 to 99%. Strains in HP4 and HP6 were 99% identical to BRAB13, isolated from a patient from the Netherlands in 2001, who was staying in a hippie community with visitors from all over the world and under primitive living conditions (45). On the other hand, the HAV strain in HP2 was closely related to strain G2B1-VP from France (98% nt identity). Therefore, all strains identified here are genotype IIA (Fig. 4E), increasing the number of completely sequenced genotype II strains to five (the other two strains are BA/ITA/2012 and CF53/Berne).

### Astroviridae.

**FIG 5:**
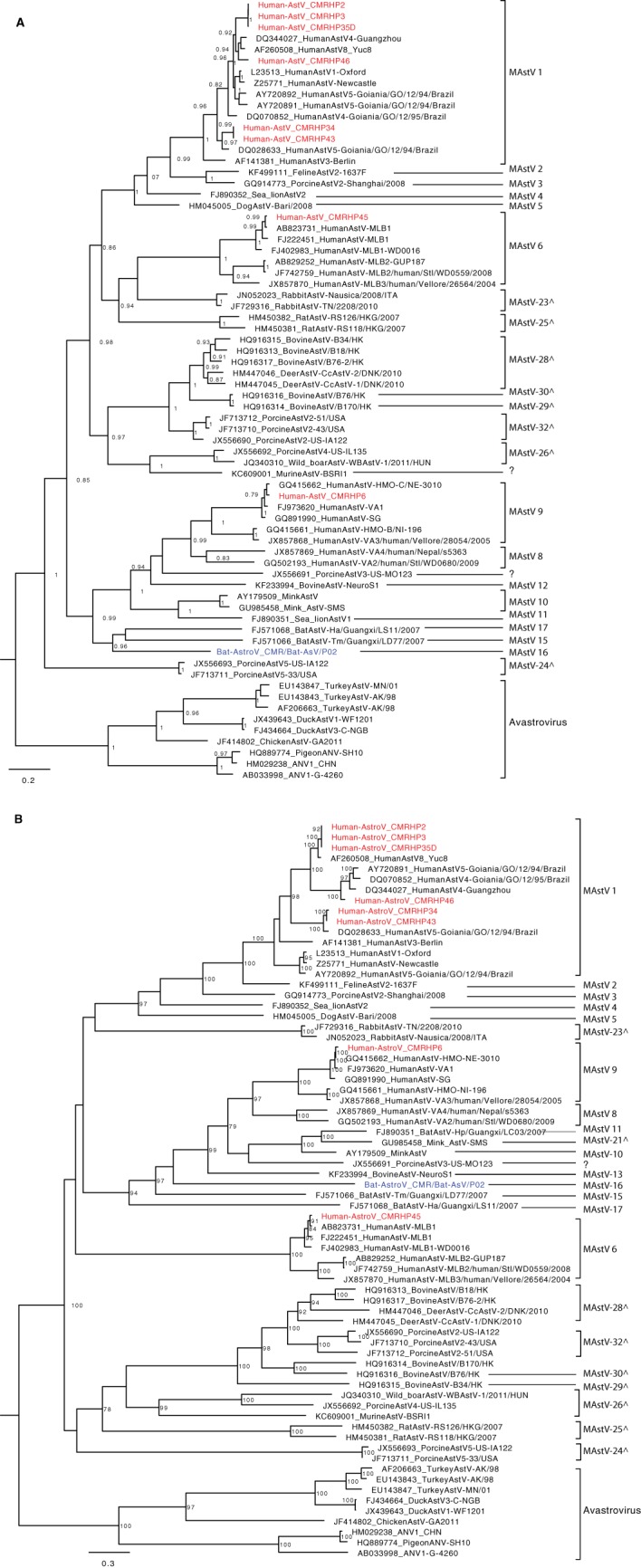
Phylogenetic trees based on the nucleotide sequences of the RdRp (A) and capsid (B) genes of the AstVs identified in this study and representative strains from GenBank. Trees were constructed using the GTR+G+I nucleotide substitution model using RAxML, with the autoMRE flag, which enables *a posteriori* bootstrapping analysis. Only bootstrap values greater than 70% are shown. Bars indicate nucleotide substitutions per site. Red, HAstVs identified in this study; blue, bat AstV strain from the same region in Cameroon; ^, proposed novel astrovirus species.

*Astroviridae* is a family of nonenveloped, spherical viruses with a linear ssRNA(+) genome of 6.8 to 7kb, containing three overlapping ORFs. The family is divided into two genera: genus *Mamastrovirus* (MAstVs) and genus *Avastrovirus* (AAstVs). The genera are further divided into 33 and 7 species, respectively (46). Fourteen out of the sixty-three human pools contained *Astroviridae* reads, and we were able to obtain eight near-complete genomes of MAstVs (HP2, 3, 6, 34, 35, 43, 45, and 46). Additionally, these pools were either from children less than 3 years old (HP2, HP45, and HP46), age group 3 to <20 (HP3, HP6, and HP35), or between 20 and <60 (HP34 and HP43). Phylogenetic analysis of the RdRp and capsid regions (Fig. 5A and B) depicted clustering of the novel MAstVs in species 1 (CMRHP2, 3, 34, 35D, 43, and 46), 6 (CMRHP45), and 9 (CMRHP6). In the MAstVs1 clade, there seems to be topological inconsistency in the different phylogenetic trees. Strain AstV8_Yuc8 (AF260508) clustered with the novel strains CMRHP2, 3, and 35D in the capsid tree, while in the RdRp tree it clustered with the Chinese strain V4-Guangzhou, suggesting a recombination event between these strains in the past. Bat astrovirus identified in Cameroon (23) formed a clade (in the RdRp tree) with other bat astroviruses from Guangxi but was distantly related to the human AstVs from the same region.

### Caliciviridae.

*Caliciviridae* are a family of nonenveloped viruses with a linear ssRNA(+) genome of 7.3 to 8.3 kb, containing two or three ORFs. The family contains five genera (47, 48). In total, *Caliciviridae* reads were found in 16 pools belonging to either the *Norovirus* or *Sapovirus* genus.

### Norovirus.

**FIG 6:**
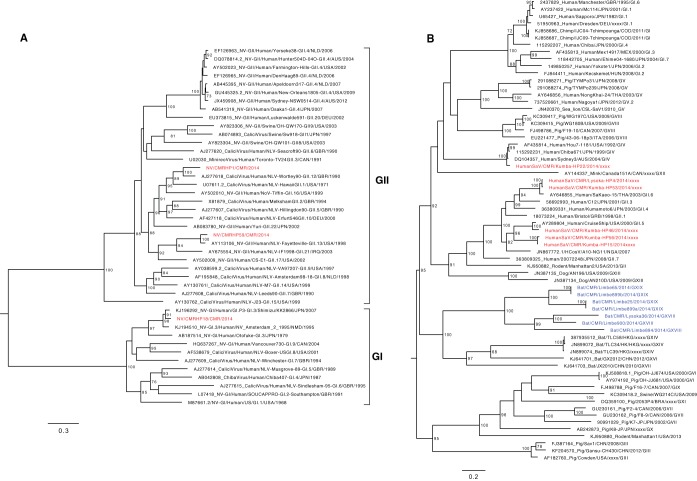
Phylogenetic relationships of representative members of the family *Caliciviridae* identified in this study: A, genus *Norovirus*; B, genus *Sapovirus*. Trees were constructed based on the nucleotide sequences of the RdRp (for norovirus) and VP1 region (for sapovirus) using the GTR+G+I substitution model using RAxML, with the autoMRE flag, which enables *a posteriori* bootstrapping analysis. Only bootstrap values greater than 70% are shown. Bars indicate nucleotide substitutions per site. Red, Cameroonian human SV strains; blue, Cameroonian bat SaVs.

This genus consists of a single species, Norwalk virus (NV), divided into 5 genogroups. Genogroups I, II, and IV infect humans, whereas genogroup III infects bovine species and genogroup V has been isolated from mice (49). Three near-complete NVs were present in the 16 pools that contained *Caliciviridae* reads (HP1, HP18, and HP59), from people who had indirect (HP1 and HP59) or no (HP18) contact with bats, and from age group A (HP1), B (HP18), or C (HP59). The phylogenetic tree (Fig. 6A) showed that the four NVs belonged to two genogroups: I (NV_CMRHP18, genotype I.3) and II (NV_CMRHP1 and NV_CMRHP59, genotypes II.12 and II.13, respectively). The novel strain NV_CMRHP18 was more than 98% similar to strain C13/2009CMR_GI.3 (a partial sequence [JF802509]) isolated from the Littoral Region of Cameroon in 2009, whereas strains of genogroup II from the same study (II.4, II.8, II.17) were distantly related to those identified here (II.12 and II.13) (7). Strains from this previous study were not included in the phylogenetic analysis because only 200 to 300 nt of the capsid region was available in databases.

### Sapovirus.

The genus *Sapovirus* (SaV) consists of a single species, Sapporo virus. It has been detected in humans, pigs, minks, dogs, sea lions, bats, chimpanzees, rodents, and carnivores (50, 51). Three near-complete SaV genomes were present in pools HP4 (age group B), HP15 (age group A), and HP22 (age group D) from people who were in indirect contact, were not in contact, and were in direct contact with bats, respectively. Phylogenetic analysis (Fig. 6B) showed that SaV from HP22 could be classified as a GIV genotype, and the SaVs HP4, HP53, HP46, HP56, and HP15 belonged to genotype GII. The phylogenetic tree showed that the bat SaVs found in Cameroon (in blue) (22) clustered together and formed a clade with other bat SaVs from China and Hong Kong but divergent from these human SaVs, indicating no evidence of interspecies transmission of SaVs in this region.

### Picobirnaviridae.

**FIG 7:**
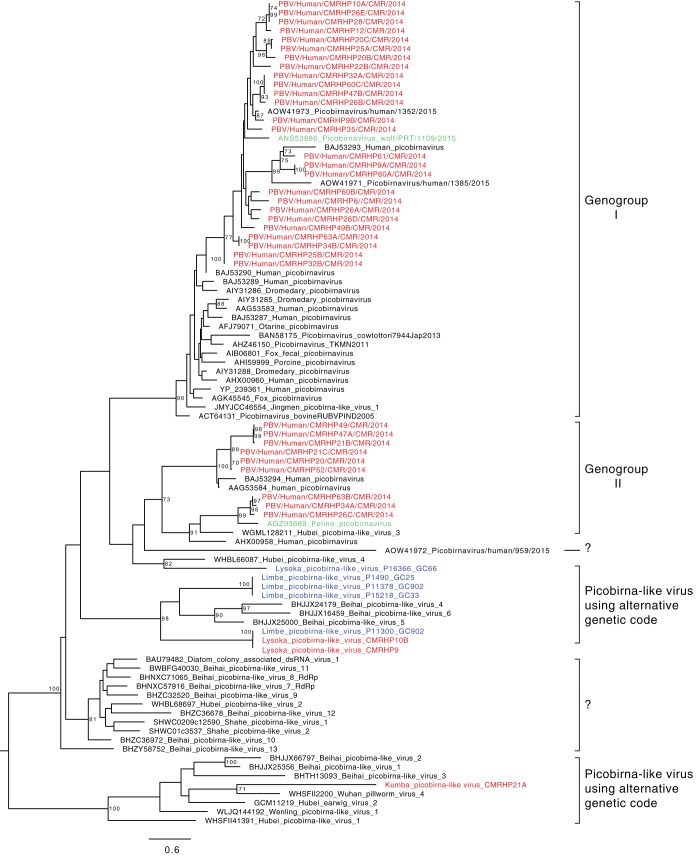
Phylogenetic relationships between PBVs isolated in this study and representative members of the family *Picobirnaviridae*, based on the amino acid sequence of the RdRp domain. The tree was constructed using the LG+G+I substitution model using RAxML, with the autoMRE flag, which enables *a posteriori* bootstrapping analysis. Only bootstrap values greater than 70% are shown. Bars indicate amino acid substitutions per site. Red, Cameroonian human PBVs; blue, Cameroonian bat PBVs; green, PBVs isolated from a wolf and a feline in Portugal.

Picobirnaviruses (PBVs) belong to the family *Picobirnaviridae*, genus *Picobirnavirus*, and are small bisegmented dsRNA viruses with a total genome size of about 4 kb. Segment one encodes a polyprotein, containing the capsid protein, and segment two encodes the RdRp. Based on the RdRp gene, PBVs are classified into two genogroups. Although PBV is genetically highly diverse and has been found in stool samples of a broad range of mammals, its true host(s) remain(s) enigmatic. The disease association is unclear, but PBV infection has been associated with gastroenteritis in both animals and humans (52, 53). Up to 28 out of the 63 pools contained reads annotated as *Picobirnaviridae* with most of the positive pools from individuals in age groups above 20. We could obtain 37 (near-complete) RdRp sequences of PBVs from these 28 pools. Phylogenetic analysis based on RdRp (Fig. 7) revealed the clustering of the novel strains in four different clades: in genogroup I (26 strains), in genogroup II (9 strains), and in 2 clades (3 strains) of uncharacterized picobirna-like viruses that use an alternative mitochondrial invertebrate genetic code (Lysoka picobirna-like virus CMRHP9 and CMRHP10B and Kumba picobirna-like virus CMRHP21A). Interestingly, a wolf PBV strain from Portugal (ANS53886) from genogroup I clustered together with human strains from Cameroon with an aa identity of 76% with strains CMRHP26A and CMRHP35. Likewise, in genogroup II, strains CMRHP34A, CMRHP63B, and CMRHP26C clustered closely (75 to 76% aa identity) with a Portuguese feline strain (AGZ93689). Intriguingly, the Cameroonian human picobirna-like viruses CMRHP9 and CMRHP10B were 99% identical to a Cameroonian bat strain picobirna-like virus, P11-300, suggesting a possible interspecies transmission. However, their true host has not yet been determined. It could be that the true hosts of PBVs are found in both humans and bats and that this therefore explains their presence in both.

### Small circular, Rep-encoding, ssDNA (CRESS-DNA) genomes. (i) Smacovirus.

**FIG 8:**
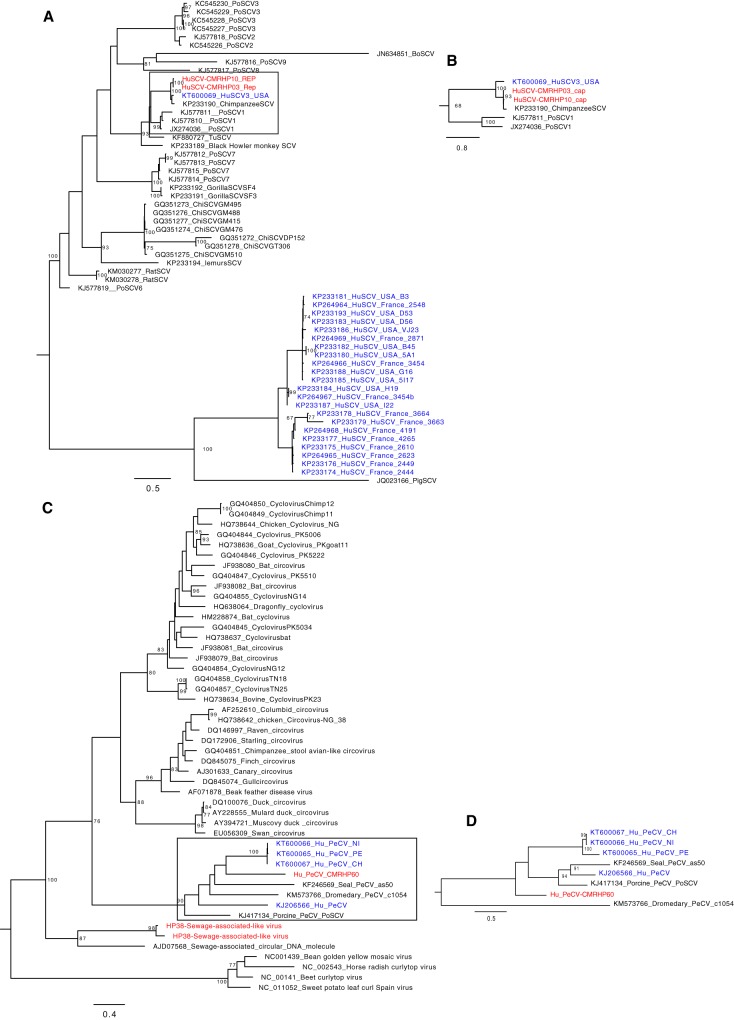
Phylogenetic tree of amino acid sequences of human and animal smacoviruses (A and B) and pecovirus (C and D) of the replicase and capsid genes, respectively. The trees were constructed using the LG+G+I substitution model using RAxML, with the autoMRE flag, which enables *a posteriori* bootstrapping analysis. Only bootstrap values greater than 70% are shown. Bars indicate amino acid substitutions per site. Red, Cameroonian human strains; blue, previously known human smacoviruses or pecovirus.

Smacovirus (SCV) is a relatively recently described virus with a small circular DNA genome with a size of about 2,529 bp. It belongs to the smacovirus group and is an unclassified eukaryotic virus of unknown origin (54). In this study, we identified two SCV sequences, one complete genome (HuSCV-CMRHP10) and a near-complete genome (HuSCV-CMRHP03). They were identified in pools of patients belonging to age group B, coming from Lysoka and having direct (HP3) or indirect (HP10) contact with bats. These strains shared 99% amino acid identity. Their replicase genes were 94% and 95% identical to chimpanzee (KP233190) and human (HuSCV3, KT600069) strains from the United States, respectively. Based on the capsid region, these novel Cameroonian strains were 98 to 99% identical to the chimp strain and only 85% identical to the human strain HuSCV3. The close genetic relatedness of human strains to a strain found in a chimpanzee sample suggest that these viruses infect a host shared between chimps and humans, and if indeed smacovirus infects mammals, this could be a case of interspecies transmission (55). Phylogenies of the replicase (Fig. 8A) and the capsid genes (Fig. 8B) indeed showed a cluster of these Cameroonian strains with a human and a chimpanzee strain from the United States. However, the topological inconsistency in the replicase and capsid trees may suggest a recombination event between these strains in the (distant) past.

### (ii) Pecovirus.

Pecoviruses (Peruvian stool-associated circo-like viruses [PeCVs]) are CRESS-DNA genomes that were first identified in the feces of a patient during an outbreak of acute gastroenteritis in the Netherlands and later in samples of Peruvian children (55, 56). Subsequently, they were identified in other humans, pigs, a dromedary camel, and a seal (55–58). Here we identified a genome sequence (HuPeCV-CMRHP60) of 2,937 bases made up of two ORFs that code for a capsid (372 aa) and a replicase protein (336 aa). Unlike other human PeCVs, the Cameroonian strain shared the same canonical nonamer (NANTATTAC) atop the predicted stem-loop structure with seal, dromedary, and porcine PeCV strains. The Rep showed 31 to 42% aa sequence identity to all other Rep genes, and a Rep-based phylogenetic analysis (Fig. 8C) showed that HuPeCV-CMRHP60 clustered together with pecovirus genomes from a seal and 3 human strains. Based on the cap protein (Fig. 8D), the Cameroonian strain was only 22 to 42% identical to all other pecoviruses and clustered only distantly from the seal strain, a porcine strain, and the human strains. This demonstrates the existence of a high level of genetic diversity within this group of circular DNA genomes, pointing to the possible existence of multiple species in this clade. Furthermore, we identified 2 incomplete sequences related to sewage-associated circular DNA molecules recovered from a sewage treatment oxidation pond in New Zealand (59), with only 38% aa identity on the Rep protein, further expanding the great diversity of CRESS-DNA genomes in the Cameroonian population.

### Bacteriophages.

Bacteriophages are viruses that infect and replicate within bacteria. Their presence therefore reflects the gut microbiota of the patients. Because most of the obtained viral reads were classified as bacteriophages, we further investigated the bacteriophage composition of the human samples. With VirSorter (60), a tool developed to identify highly divergent dsDNA phages from metagenomics data, 5,905 of the contigs in our data set were identified as phages. From these, the tool Diamond (61) annotated 2,647 as bacterial, 21 as metazoan, and only 606 (∼10%) as viral, while 1,309 contigs remained unannotated. From the contigs annotated as viral by Diamond, most were phages belonging to the *Myoviridae* (236 contigs), *Podoviridae* (95 contigs), *Siphoviridae* (145 contigs), and *Microviridae* (36 contigs) families. To get insight into the differences in the bacteriophage communities, we compared the VirSorter-identified bacteriophage richness between the different age groups (Fig. S6A), locations (Fig. S6B,) and bat contact status (Fig. S6C), all of which showed no significant differences. To identify the potential bacterial hosts of these phages, we searched for bacterial CRISPR spacer sequences in the phage contigs, to identify its potential host. The search revealed that the most likely hosts of these phages are bacteria of the families *Bacteroidaceae*, *Bifidobacteriaceae*, *Enterobacteriaceae*, *Enterococcaceae*, *Erysipelotrichaceae*, *Eubacteriaceae*, *Lactobacillaceae*, *Odoribacteraceae*, *Streptococcaceae*, and *Veillonellaceae* (Table S2).

10.1128/mSphere.00585-18.6 FIG S6 Phageome richness comparisons between (A) different age groups, (B) different locations, and (C) bat contact status. Download FIG S6, PDF file, 0.10 MB. Copyright © 2019 Yinda et al. 2019 Yinda et al. This content is distributed under the terms of the Creative Commons Attribution 4.0 International license.

10.1128/mSphere.00585-18.9 TABLE S2 Results of CRISPR sequence BLASTn against the phage community revealing a diverse bacterial community. Download Table S2, PDF file, 0.1 MB. Copyright © 2019 Yinda et al. 2019 Yinda et al. This content is distributed under the terms of the Creative Commons Attribution 4.0 International license.

### Network analysis of human and bat phageomes.

**FIG 9:**
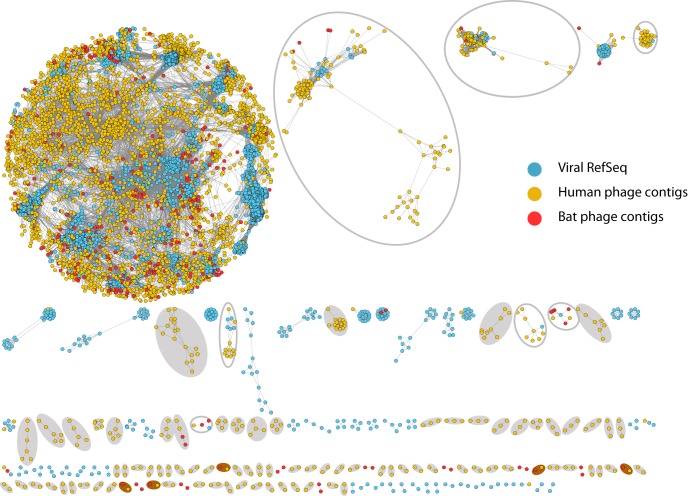
Network analysis of the phageome of the bat and human pools with the prokaryotic and archaeal viral RefSeq viruses (in light sky blue). In yellow and red are viral contigs shown identified in Cameroonian humans and bat samples, respectively. Gray filled ovals, clusters containing only novel phages; gray open ovals, clusters where a large fraction of the phages were identified in this study; brown filled ovals, clusters of novel bat and human phages only.

In order to visualize the genetic relatedness between the human and bat gut phageome, a recently developed bioinformatics tool (vConTACT) was used. It groups phages based on their genome sequences into viral clusters which correlate rather well with viral genera as defined by the International Committee of Taxonomy of Viruses (ICTV) (62). A total of 30,875 protein clusters were predicted using the prokaryotic and archaeal RefSeq combined with the proteins predicted from the phage contigs identified from the human and bats pools using VirSorter. Using a network analysis approach (Fig. 9), 792 viral genome clusters were predicted of which 173 contained reference phages together with bat or human phage contigs, whereas the rest contained only bat, only human, or bat-human clusters. Figure 9 shows that both Cameroonian human and bat phage contigs identified in our studies are spread across the known phage sequence space. However, several of the phage contigs constituted completely new clusters (indicated by filled gray ovals), completely unconnected to phages in the reference database. Also, the genetic diversity of several previously known phage subclusters was significantly expanded (as indicated by open ovals) while some clusters (in brown ovals) were made up of only bat and human phages identified in this study.

## DISCUSSION

Recently, we thoroughly investigated the gut virome of fruit bats from Cameroon (20–23, 63) and showed the presence of many novel and divergent eukaryotic viral families, including viruses known to cause gastroenteritis in humans. The aim of the current study was to investigate the gut virome of humans (*n* = 221) from Cameroon and to further determine if bat viruses are possible causative agents of gastrointestinal infections in humans.

Twenty-four percent of the 471 million generated trimmed reads were assigned as viral. Most of these reads were bacteriophages, which is in accordance with previous studies (64). The eukaryotic viral reads include those that belong to viral families that are commonly associated with gastroenteritis in humans (*Adenoviridae*, *Astroviridae*, *Caliciviridae*, *Picornaviridae*, and *Reoviridae*), viruses that are uncommon causes of gastroenteritis (orthoreovirus), or those that have been identified in humans but not associated with disease (anellovirus, smacovirus, and picrobirna-like viruses).

### Common human gastroenteric viruses: *Picornaviridae* and *rotavirus A*.

Among the viruses known to cause gastroenteric disease, reads belonging to the *Picornaviridae* family were identified most frequently (Fig. 1B). This is partly because it is one of the largest viral families and is made up of at least 29 genera, many of which are transmitted through the fecal-oral or respiratory route (28). Most of these infections were in pools of individuals less than 20 years of age (Fig. 1C). This finding is consistent with previous findings from Cameroon, where a high prevalence of EV in children was reported using PCR-based approaches (65). Furthermore, most of the EVs here were of genotype C, also supporting a recent study that identified a high rate of EV Cs in the northern regions of Cameroon (31). Therefore, the high prevalence of EV C is probably national. However, the absence of genetic relatedness between the Cameroonian human EV strains and animal strains (from chimp and gorilla [66]) does not indicate interspecies transmission of EVs from animals. Additionally, we report for the first time (nearly) complete genomes of picornaviruses of the genera *Parechovirus*, *Cardiovirus*, *Hepatovirus A*, and *Cosavirus* from Cameroonian patients. This broadens the range of picornaviruses found in the Cameroonian population, indicating that picornaviruses might be playing a vital role in gastroenteric viral infection in the Cameroonian population, especially given that most of these were from samples of sick children.

Rotavirus A (RVA), a common viral gastroenteritis-causing agent, was identified only in a limited number of pools. This was previously observed in Cameroon, and possible reasons for the low prevalence could include the acute nature of rotavirus infections or seasonal changes in rotavirus infections (6). Of note, rotavirus vaccination was introduced in Cameroon in April 2014, coinciding with the period of sample collection of this study (February to September 2014); however, the vaccination campaign had not started in the sampling locations within this period, and therefore, the result represents a prevaccination rotavirus prevalence status. The identified rotavirus strain showed 3% nt differences with the vaccine strain, further suggesting that this was a wild-type RVA strain, rather than a vaccine-derived strain.

### Uncommon human gastroenteric virus: mammalian orthoreovirus (MORV).

This first MORV strain from Cameroon showed topological incongruence in its phylogeny, thereby pointing to possible reassortment events in the past. The phylogenetic clustering of some segments to strains from animals (rodents and bats) could be an indication of a zoonotic event or could also be due to the absence of related strains in databases from an unknown host. Given that this strain was from a pool of samples from two children suffering from severe diarrhea, it is not unlikely that this strain might have contributed to the disease. Therefore, MORV might be playing a greater role in diarrheal diseases in this region than was previously known. Hence, extensive epidemiological studies in different regions and in different hosts are required to fully delineate the prevalence, genomics, and interspecies transmissibility of MORV.

### Viruses not (yet) associated with gastroenteritis: *Picobirnaviridae*, smacovirus, and *Anelloviridae.*

Apart from the above-mentioned gastroenteritis-related viruses, several other viruses with unelucidated gastroenteric roles were also identified in this study. First, we observed fewer reads of picobirnaviruses (PBVs) in pools from children than in adults. Previous studies also detected a relatively low percentage of children with PBVs (67, 68). This therefore adds up to the notion that PBVs are likely to be absent in infants and young children and only start to increase with age and potentially a changing diet, though this needs to be further proven (69, 70). Interestingly, the genetic relatedness of a human picobirna-like virus with one that was found in a bat pool from the same region suggests an interspecies transmission. However, these picobirna-like sequences are translated using an alternative mitochondrial codon, indicating that their hosts may not be mammals. A principal component analysis of the codon usage bias of different known mitochondrial genome sequences, mitoviruses, and PBVs seems to suggest that they may have the same lifestyle as mitoviruses known to infect fungal mitochondria (71). However, the recent identification of a bacterial ribosomal binding site in PBV genomes suggests prokaryotes as a potential host (72). Given that the mitochondria have descended from ancient eubacterial endosymbionts (73), this may explain the clustering of these PBVs with mitoviruses. Therefore, the question about the true host of PBVs remains controversial.

Second, for the first time, two strains of African smacovirus (SCV) were identified in Cameroonian samples. Their genetic relatedness to a chimpanzee strain (isolated from a captive chimp in a zoo in San Francisco) and a strain from a child from the United States (54, 55) indicates either an interspecies transmission event or the presence of a shared viral host in both humans and chimps. Although the role of smacovirus in gastroenteritis has not been elucidated, their presence in cases of unexplained diarrhea in French patients seems to indicate a potential role in gastroenteritis (54); hence, these could be instances of interspecies transmissions.

The percentages of pools positive for anelloviruses were higher in age categories of children and the old and lower for the middle-aged groups. Given the well-established notion that infants and the elderly have reduced immunity (74, 75), this could be in line with previous studies that suggest a link between the burden of anelloviruses and host immune competence (76–78). Despite their ubiquity, *Anelloviridae* have an undefined implication in hosts’ health and are thought to be probably asymptomatic (harmless) or even beneficial. However, they have been associated with hepatitis, pulmonary diseases, hematologic disorders, myopathy, and lupus, but it is not clear if their presence is the cause or the result of disease progression (79–82).

### Human viruses and interspecies transmission from bats.

In bats from the same area, we were able to identify gastroenteritis-related and nonrelated viruses. Here, the corresponding viruses identified from the families *Astroviridae* (astrovirus), *Caliciviridae* (sapovirus), and *Reoviridae* (RVA) are genetically diverse from those identified in bats from the same region, indicating no evidence of recent interspecies transmissions between bats and humans (63). However, genetic relatedness of human MORV to animal strains showed the possibility of zoonosis between humans and not only bats but animals in general. Additionally, the presence of some Cameroonian strains of SCV and PBV in bats or other animals would indicate interspecies transmissions if their infectivity in these animals is fully elucidated.

### Human and bat phageome.

In this study, we detected a huge phage community with a great diversity beyond the range of known bacteriophages in reference databases, potentially representing the gut microbiome diversity in the patients (83, 84). Overall, this further supports the idea that the full phageome richness is still to be completely elucidated (85). Furthermore, network analysis indicates the presence of completely novel phage groups and that phage genera in the gut microbiota might be shared between humans and bats.

### Conclusion.

Several diverse viruses were discovered in the gut virome of Cameroonians. Some of these were already known to be the causative agent of gastroenteritis, whereas others are likely to be the cause of gastroenteric problems in the patients. Further screening of patients for these viruses will be needed to establish their prevalence in the population, allowing for more appropriate measures and treatment and prevention of viral gastroenteritis. Also, to be able to completely elucidate the role of the novel viruses like pecovirus and smacovirus, more studies are required. Further attention should also be given to newly identified viruses (for example, MORV) and their potential as emerging pathogens in the human population.

## MATERIALS AND METHODS

### Ethical authorization.

Ethical authorization for the use of human samples was obtained from the Cameroon National Ethics Committee, Yaoundé. All human experiments were performed in accordance with the Ministry’s National Ethics Committee guidelines.

### Sample collection and preparation.

Human fecal samples were collected between February and September 2014, after informed consent was obtained from patients in two different hospitals (Lysoka Health District and Kumba District Hospital of the Southwest region of Cameroon). This region was chosen because here bats are hunted, sold, and eaten. Diarrheic patients and/or people who came into contact with bats directly (by eating, hunting, or handling) or indirectly (if a family member was directly exposed to bats) were eligible for sampling. A total of 221 samples were collected from subjects between age 0 and <3 years (age group A, 80 samples), 3 and <20 (age group B, 63 samples), 20 and <60 (age group C, 65 samples), and 60 and older (age group D, 13 samples). All the samples were from people who had symptoms of gastroenteritis, except 2 from age group C who had contact with bats. Samples were then placed into labeled tubes containing universal transport medium (UTM), placed on dry ice, and stored at −20°C, until being shipped to the Laboratory of Viral Metagenomics, Leuven, Belgium. The samples were stored at −80°C until used (63).

Fecal samples were first diluted using UTM, and equal volumes of the dilutions were pooled based on the location, age, and bat contact status (direct, indirect, or none). Each pool contained two to five samples, and for the different age groups (A to D) we had 22, 17, 20, and 4 pools, respectively. The pools were then treated according to the NetoVIR protocol (86). Briefly, the pools (10% [wt/vol] fecal suspensions) were homogenized for 1 min at 3,000 rpm with a Minilys homogenizer (Bertin Technologies) and filtered using an 0.8-μm PES filter (Sartorius). The filtrate was then treated with a cocktail of Benzonase (Novagen) and micrococcal nuclease (New England Biolabs) at 37°C for 2 h to digest free-floating nucleic acids. Total nucleic acids (both RNA and DNA) were extracted using the QIAamp viral RNA minikit (Qiagen) according to the manufacturer’s instructions but without addition of carrier RNA to the lysis buffer. First- and second-strand synthesis and random PCR amplification for 17 cycles were performed using a slightly modified whole-transcriptome amplification (WTA2) kit procedure (Sigma-Aldrich). WTA2 products were purified with MSB Spin PCRapace spin columns (Stratec), and the libraries were prepared for Illumina sequencing using a slightly modified version of the Nextera XT library preparation kit (Illumina), which is described in detail in reference 86. Samples were pooled in an attempt to obtain an average of approximately 10 million paired-end reads per pool. Sequencing was performed on a NextSeq 500 high-output platform (Illumina) for 300 cycles (2 × 150-bp paired ends).

### Genomic and phylogenetic analysis.

NGS reads were analyzed as described in the work of Yinda et al. (20, 63). Briefly, raw reads were trimmed using Trimmomatic (parameters: HEADCROP:19 LEADING:15 TRAILING:15 SLIDINGWINDOW:4:20 MINLEN:50) and FastUniq to remove identical reads. The *de novo* assembly or reads and annotation of reads were performed using SPAdes (with the meta flag) and Diamond (with the sensitive option using the GenBank nonredundant database), respectively (61, 87, 88). Open reading frames (ORFs) of contigs of interest were identified and further analyzed for conserved motifs in the amino acid sequences using NCBI’s conserved domain database (CDD) (89). Nucleotide and amino acid alignments of viral sequences were done with MUSCLE implemented in MEGA7 (90) or MAFFT (91). Substitution models were determined using ModelGenerator (92), and phylogenetic trees were constructed using RAxML (93), with the autoMRE flag, which enables *a posteriori* bootstrapping analysis. All trees were visualized in FigTree (http://tree.bio.ed.ac.uk/software/figtree/) and midpoint rooted for purposes of clarity.

### Phageome analysis.

Contig annotation with DIAMOND is dependent on the accuracy of the database used, and in most databases, phages are poorly annotated. However, VirSorter uses a manually curated database of virus reference genomes augmented with metagenomic viral sequences sampled from freshwater, seawater, and human gut, lung, and saliva. Hence, for further identification of bacteriophages, scaffolds >1 kb were classified using VirSorter (decontamination mode [60]). Only scaffolds assigned to categories 1 and 2 were considered bacteriophage contigs and were filtered for redundancy at 95% nucleotide identity over 70% of the length using Cluster Genomes (94). Then, trimmed reads from each pool were mapped using Bowtie 2 (95) to the bacteriophage contigs, and the generated BAM files were filtered to remove reads that aligned at <95% identity using BamM (http://ecogenomics.github.io/BamM/). Abundance tables were obtained and normalized for total number of reads of each sample. For the richness comparison, Mann-Whitney tests were used, and for the clustering, an Adonis test was performed. All downstream analyses were done in R (96) using the vegan package (97). Furthermore, to identify the potential corresponding bacterial host, a database of these contigs was made to which a nucleotide BLASTN search (100% identity without gaps) was performed using a fasta file of CRISPR sequences (98) as query. These sequences correspond to different bacterial hosts, and their presence in the phage genome highlight the potential host of the phage.

To see if the phage community of these humans is related to those of the bats from the same locality, a visualization of the network of both human and bat phageomes was performed using vConTACT (62). Initially, proteins were predicted using Prodigal (99), and combined with the Viral RefSeq of archaeal and prokaryotic predicted proteins. A database was generated from the contigs of bat pools, human pools, and viral RefSeq proteins, and BLASTp was performed against the combined proteins. The output of blast was used to run vConTACT, and the output network was visualized in Cytoscape (100).

### Data availability.

All sequences were deposited in GenBank under the following accession numbers: MH608285 to MH608287 and MH933752 to MH933860 (details in Table S3). Raw reads were submitted to the NCBI’s Short Read Archive (SRA) under the project ID PRJNA491626.

10.1128/mSphere.00585-18.7 TABLE S3 (A) Accession numbers of all viruses described in the study. (B) Accession numbers of raw reads from individual pools submitted to Short Read Archive under the BioProject number PRJNA491626 and Tax ID 1861841. For ethical reasons, reads mapping to the human genome were removed. Download Table S3, PDF file, 0.1 MB. Copyright © 2019 Yinda et al. 2019 Yinda et al. This content is distributed under the terms of the Creative Commons Attribution 4.0 International license.

## References

[1] World Health Organization. 2017 Global Health Observatory data repository. GHO | World—diarrhoeal diseases. World Health Organization, Geneva, Switzerland.

[2] WHO. 2015 Levels and trends in child mortality 2015. World Health Organization, Geneva, Switzerland.

[3] Fong T-T, Lipp EK 2005 Enteric viruses of humans and animals in aquatic environments: health risks, detection, and potential water quality assessment tools. Microbiol Mol Biol Rev 69:357–371. doi:10.1128/MMBR.69.2.357-371.2005. 15944460 PMC1197419

[4] Ayukekbong J, Kabayiza J-C, Lindh M, Nkuo-Akenji T, Tah F, Bergström T, Norder H 2013 Shift of Enterovirus species among children in Cameroon; identification of a new enterovirus, EV-A119. J Clin Virol 58:227–232. doi:10.1016/j.jcv.2013.07.005. 23895932

[5] Santosham M, Chandran A, Fitzwater S, Fischer-Walker C, Baqui AH, Black R 2010 Progress and barriers for the control of diarrhoeal disease. Lancet 376:63–67. doi:10.1016/S0140-6736(10)60356-X. 20609988

[6] Ayukekbong JA, Andersson ME, Vansarla G, Tah F, Nkuo-AkenjI T, Lindh M, Bergström T 2014 Monitoring of seasonality of norovirus and other enteric viruses in Cameroon by real-time PCR: an exploratory study. Epidemiol Infect 142:1393–1402. doi:10.1017/S095026881300232X. 24047516 PMC9151225

[7] Ayukekbong J, Lindh M, Nenonen N, Tah F, Nkuo-Akenji T, Bergström T 2011 Enteric viruses in healthy children in Cameroon: viral load and genotyping of norovirus strains. J Med Virol 83:2135–2142. doi:10.1002/jmv.22243. 22012721

[8] Ayukekbong JA 2013 PhD thesis. University of Gothenburg, Gothenburg, Sweden.

[9] Li L, Victoria J, Kapoor A, Blinkova O, Wang C, Babrzadeh F, Mason CJ, Pandey P, Triki H, Bahri O, Oderinde BS, Baba MM, Bukbuk DN, Besser JM, Bartkus JM, Delwart EL 2009 A novel picornavirus associated with gastroenteritis. J Virol 83:12002–12006. doi:10.1128/JVI.01241-09. 19759142 PMC2772710

[10] Phan TG, Sdiri-Loulizi K, Aouni M, Ambert-Balay K, Pothier P, Deng X, Delwart E 2014 New parvovirus in child with unexplained diarrhea, Tunisia. Emerg Infect Dis 20:1911–1913. doi:10.3201/eid2011.140428. 25340816 PMC4214302

[11] Bosch A, Guix S, Sano D, Pintó RM 2008 New tools for the study and direct surveillance of viral pathogens in water. Curr Opin Biotechnol 19:295–301. doi:10.1016/j.copbio.2008.04.006. 18508257 PMC7126527

[12] Fenton M, Simmons N 2015 Bats, a world of science and mystery. The University of Chicago Press, Chicago, IL.

[13] Rupprecht CE, Smith JS, Fekadu M, Childs JE 1995 The ascension of wildlife rabies: a cause for public health concern or intervention? Emerg Infect Dis 1:107–114. doi:10.3201/eid0104.950401. 8903179 PMC2626887

[14] Towner JS, Pourrut X, Albariño CG, Nkogue CN, Bird BH, Grard G, Ksiazek TG, Gonzalez J-P, Nichol ST, Leroy EM 2007 Marburg virus infection detected in a common African bat. PLoS One 2:e764. doi:10.1371/journal.pone.0000764. 17712412 PMC1942080

[15] Leroy EM, Kumulungui B, Pourrut X, Rouquet P, Hassanin A, Yaba P, Delicat A, Paweska JT, Gonzalez J-P, Swanepoel R 2005 Fruit bats as reservoirs of Ebola virus. Nature 438:575–576. doi:10.1038/438575a. 16319873

[16] Lau SKP, Woo PCY, Li KSM, Huang Y, Tsoi H-W, Wong BHL, Wong SSY, Leung S-Y, Chan K-H, Yuen K-Y 2005 Severe acute respiratory syndrome coronavirus-like virus in Chinese horseshoe bats. Proc Natl Acad Sci U S A 102:14040–14045. doi:10.1073/pnas.0506735102. 16169905 PMC1236580

[17] Memish ZA, Mishra N, Olival KJ, Fagbo SF, Kapoor V, Epstein JH, AlHakeem R, Durosinloun A, Al Asmari M, Islam A, Kapoor A, Briese T, Daszak P, Al Rabeeah AA, Lipkin WI 2013 Middle East respiratory syndrome coronavirus in bats, Saudi Arabia. Emerg Infect Dis 19:1819–1823. doi:10.3201/eid1911.131172. 24206838 PMC3837665

[18] Chua KB, Lek Koh C, Hooi PS, Wee KF, Khong JH, Chua BH, Chan YP, Lim ME, Lam SK 2002 Isolation of Nipah virus from Malaysian Island flying-foxes. Microbes Infect 4:145–151. 11880045 10.1016/s1286-4579(01)01522-2

[19] Wong S, Lau S, Woo P, Yuen K-YY 2007 Bats as a continuing source of emerging infections in humans. Rev Med Virol 17:67–91. doi:10.1002/rmv.520. 17042030 PMC7169091

[20] Yinda CK, Rector A, Zeller M, Conceição-Neto N, Heylen E, Maes P, Stephen GM, Van Ranst M, Matthijnssens J, Ghogomu SM, Van Ranst M, Matthijnssens J 2016 A single bat species in Cameroon harbors multiple highly divergent papillomaviruses in stool identified by metagenomics analysis. Virol Rep 6:74–80. doi:10.1016/j.virep.2016.08.001. PMC7103942 32289018

[21] Yinda CK, Zell R, Deboutte W, Zeller M, Conceição-Neto N, Heylen E, Maes P, Knowles NJ, Ghogomu SM, Van Ranst M, Matthijnssens J 2017 Highly diverse population of Picornaviridae and other members of the Picornavirales, in Cameroonian fruit bats. BMC Genomics 18:249. doi:10.1186/s12864-017-3632-7. 28335731 PMC5364608

[22] Yinda CK, Conceição-Neto N, Zeller M, Heylen E, Maes P, Ghogomu SM, Van Ranst M, Matthijnssens J 2017 Novel highly divergent sapoviruses detected by metagenomics analysis in straw-colored fruit bats in Cameroon. Emerg Microbes Infect 6:e38. doi:10.1038/emi.2017.20. 28536431 PMC5520483

[23] Yinda CK, Ghogomu SM, Conceição-Neto N, Beller L, Deboutte W, Vanhulle E, Maes P, Van Ranst M, Matthijnssens J 2018 Cameroonian fruit bats harbor divergent viruses, including rotavirus H, bastroviruses, and picobirnaviruses using an alternative genetic code. Virus Evol 4:vey008. doi:10.1093/ve/vey008. 29644096 PMC5888411

[24] Steyer A, Gutiérrez-Aguire I, Kolenc M, Koren S, Kutnjak D, Pokorn M, Poljšak-Prijatelj M, Racki N, Ravnikar M, Sagadin M, Fratnik Steyer A, Toplak N 2013 High similarity of novel orthoreovirus detected in a child hospitalized with acute gastroenteritis to mammalian orthoreoviruses found in bats in Europe. J Clin Microbiol 51:3818–3825. doi:10.1128/JCM.01531-13. 24025904 PMC3889772

[25] Lelli D, Moreno A, Lavazza A, Bresaola M, Canelli E, Boniotti MB, Cordioli P 2013 Identification of mammalian orthoreovirus type 3 in Italian bats. Zoonoses Public Health 60:84–92. doi:10.1111/zph.12001. 22931153

[26] Ciarlet M, Estes M 2002 Rotaviruses: basic biology, epidemiology and methodologies, p 2573–2773. *In* Britton G (ed), Encyclopedia of environmental microbiology, John Wiley & Sons, New York, NY.

[27] Estes MK, Cohen J 1989 Rotavirus gene structure and function. Microbiol Rev 53:410–449. 2556635 10.1128/mr.53.4.410-449.1989 PMC372748

[28] Knowles NJ, Hovi T, Hyypiä T, King AMQ, Lindberg M, Pallansch MA, Palmenberg AC, Simmonds P, Skern T, Stanway G, Yamashita T, Zell R 2012 Picornaviridae, p 855–880. *In* King AMQ, Adams MJ, Carstens EB, Lefkowitz EJ (ed), Virus taxonomy: classification and nomenclature of viruses. Ninth report of the International Committee on Taxonomy of Viruses. Elsevier, San Diego, CA.

[29] Tracy S, Chapman NM, Drescher KM, Kono K, Tapprich W 2006 Evolution of virulence in picornaviruses, p 193–209. *In* Domingo E (ed), Quasispecies: concept and implications for virology. Springer, Berlin, Germany. 10.1007/3-540-26397-7_7 16568900

[30] Wang X, Ren J, Gao Q, Hu Z, Sun Y, Li X, Rowlands DJ, Yin W, Wang J, Stuart DI, Rao Z, Fry EE 2015 Hepatitis A virus and the origins of picornaviruses. Nature 517:85–88. doi:10.1038/nature13806. 25327248 PMC4773894

[31] Sadeuh-Mba SA, Bessaud M, Massenet D, Joffret M-L, Endegue M-C, Njouom R, Reynes J-M, Rousset D, Delpeyroux F 2013 High frequency and diversity of species C enteroviruses in Cameroon and neighboring countries. J Clin Microbiol 51:759–770. doi:10.1128/JCM.02119-12. 23254123 PMC3592076

[32] Kroneman A, Vennema H, Deforche K, Avoort HVD, Peñaranda S, Oberste MS, Vinjé J, Koopmans M 2011 An automated genotyping tool for enteroviruses and noroviruses. J Clin Virol 51:121–125. doi:10.1016/j.jcv.2011.03.006. 21514213

[33] Tolf C, Gullberg M, Johansson ES, Tesh RB, Andersson B, Lindberg AM 2009 Molecular characterization of a novel Ljungan virus (Parechovirus; Picornaviridae) reveals a fourth genotype and indicates ancestral recombination. J Gen Virol 90:843–853. doi:10.1099/vir.0.007948-0. 19264646 PMC2889435

[34] Sun G, Wang Y, Tao G, Shen Q, Cao W, Chang X, Zhang W, Shao C, Yi M, Shao S, Yang Y 2012 Complete genome sequence of a novel type of human parechovirus strain reveals natural recombination events. J Virol 86:8892–8893. doi:10.1128/JVI.01241-12. 22843855 PMC3421728

[35] Figueroa JP, Ashley D, King D, Hull B 1989 An outbreak of acute flaccid paralysis in Jamaica associated with echovirus type 22. J Med Virol 29:315–319. 2621458 10.1002/jmv.1890290418

[36] Koskiniemi M, Paetau R, Linnavuori K 1989 Severe encephalitis associated with disseminated echovirus 22 infection. Scand J Infect Dis 21:463–466. 2587949 10.3109/00365548909167453

[37] Oberste MS, Maher K, Kilpatrick DR, Pallansch MA 1999 Molecular evolution of the human enteroviruses: correlation of serotype with VP1 sequence and application to picornavirus classification. J Virol 73:1941–1948. 9971773 10.1128/jvi.73.3.1941-1948.1999 PMC104435

[38] Chuchaona W, Khamrin P, Yodmeeklin A, Saikruang W, Kongsricharoern T, Ukarapol N, Okitsu S, Hayakawa S, Ushijima H, Maneekarn N 2015 Detection and characterization of a novel human parechovirus genotype in Thailand. Infect Genet Evol 31:300–304. doi:10.1016/j.meegid.2015.02.003. 25681699

[39] Holtz LR, Finkbeiner SR, Kirkwood CD, Wang D 2008 Identification of a novel picornavirus related to cosaviruses in a child with acute diarrhea. Virol J 5:159. doi:10.1186/1743-422X-5-159. 19102772 PMC2615758

[40] Naeem A, Hosomi T, Nishimura Y, Alam MM, Oka T, Zaidi SSZ, Shimizu H 2014 Genetic diversity of circulating Saffold viruses in Pakistan and Afghanistan. J Gen Virol 95:1945–1957. doi:10.1099/vir.0.066498-0. 24899154

[41] Chiu CY, Greninger AL, Kanada K, Kwok T, Fischer KF, Runckel C, Louie JK, Glaser CA, Yagi S, Schnurr DP, Haggerty TD, Parsonnet J, Ganem D, DeRisi JL 2008 Identification of cardioviruses related to Theiler’s murine encephalomyelitis virus in human infections. Proc Natl Acad Sci U S A 105:14124–14129. doi:10.1073/pnas.0805968105. 18768820 PMC2528868

[42] Ren L, Gonzalez R, Xiao Y, Xu X, Chen L, Vernet G, Paranhos-Baccalà G, Jin Q, Wang J 2009 Saffold cardiovirus in children with acute gastroenteritis, Beijing, China. Emerg Infect Dis 15:1509–1511. doi:10.3201/eid1509.081531. 19788828 PMC2819865

[43] Cristina J, Costa-Mattioli M 2007 Genetic variability and molecular evolution of hepatitis A virus. Virus Res 127:151–157. doi:10.1016/j.virusres.2007.01.005. 17328982

[44] Franco E, Meleleo C, Serino L, Sorbara D, Zaratti L 2012 Hepatitis A: epidemiology and prevention in developing countries. World J Hepatol 4:68–73. doi:10.4254/wjh.v4.i3.68. 22489258 PMC3321492

[45] Tjon GMS, Wijkmans CJ, Coutinho RA, Koek AG, van den Hoek JAR, Leenders ACAP, Schneeberger PM, Bruisten SM 2005 Molecular epidemiology of hepatitis A in Noord-Brabant, The Netherlands. J Clin Virol 32:128–136. doi:10.1016/j.jcv.2004.03.008. 15653415

[46] Donato C, Vijaykrishna D 2017 The broad host range and genetic diversity of mammalian and avian astroviruses. Viruses 9:102. doi:10.3390/v9050102. PMC5454415 28489047

[47] Xi JN, Graham DY, Wang KN, Estes MK 1990 Norwalk virus genome cloning and characterization. Science 250:1580–1583. 2177224 10.1126/science.2177224

[48] McFadden N, Bailey D, Carrara G, Benson A, Chaudhry Y, Shortland A, Heeney J, Yarovinsky F, Simmonds P, Macdonald A, Goodfellow I 2011 Norovirus regulation of the innate immune response and apoptosis occurs via the product of the alternative open reading frame 4. PLoS Pathog 7:e1002413. doi:10.1371/journal.ppat.1002413. 22174679 PMC3234229

[49] Zheng D-P, Ando T, Fankhauser RL, Beard RS, Glass RI, Monroe SS 2006 Norovirus classification and proposed strain nomenclature. Virology 346:312–323. doi:10.1016/j.virol.2005.11.015. 16343580

[50] Oka T, Lu Z, Phan T, Delwart EL, Saif LJ, Wang Q 2016 Genetic characterization and classification of human and animal sapoviruses. PLoS One 11:e0156373. doi:10.1371/journal.pone.0156373. 27228126 PMC4881899

[51] Olarte-Castillo XA, Hofer H, Goller KV, Martella V, Moehlman PD, East ML 2016 Divergent sapovirus strains and infection prevalence in wild carnivores in the Serengeti ecosystem: a long-term study. PLoS One 11:e0163548. doi:10.1371/journal.pone.0163548. 27661997 PMC5035092

[52] Conceicao-Neto N, Mesquita JR, Zeller M, Yinda CK, Álvares F, Roque S, Petrucci-Fonseca F, Godinho R, Heylen E, Van Ranst M, Matthijnssens J 2016 Reassortment among picobirnaviruses found in wolves. Arch Virol 161:2859–2862. doi:10.1007/s00705-016-2987-4. 27438074

[53] Malik YS, Kumar N, Sharma K, Dhama K, Shabbir MZ, Ganesh B, Kobayashi N, Banyai K 2014 Epidemiology, phylogeny, and evolution of emerging enteric Picobirnaviruses of animal origin and their relationship to human strains. Biomed Res Int 2014:780752. doi:10.1155/2014/780752. 25136620 PMC4124650

[54] Ng TFF, Zhang W, Sachsenröder J, Kondov NO, da Costa AC, Vega E, Holtz LR, Wu G, Wang D, Stine CO, Antonio M, Mulvaney US, Muench MO, Deng X, Ambert-Balay K, Pothier P, Vinjé J, Delwart E 2015 A diverse group of small circular ssDNA viral genomes in human and non-human primate stools. Virus Evol 1:vev017. doi:10.1093/ve/vev017. 27774288 PMC5014484

[55] Phan TG, da Costa AC, del Valle Mendoza J, Bucardo-Rivera F, Nordgren J, O’Ryan M, Deng X, Delwart E 2016 The fecal virome of South and Central American children with diarrhea includes small circular DNA viral genomes of unknown origin. Arch Virol 161:959–966. doi:10.1007/s00705-016-2756-4. 26780893 PMC4814309

[56] Smits SL, Schapendonk CME, van Beek J, Vennema H, Schürch AC, Schipper D, Bodewes R, Haagmans BL, Osterhaus ADME, Koopmans MP 2014 New viruses in idiopathic human diarrhea cases, the Netherlands. Emerg Infect Dis 20:1218–1222. doi:10.3201/eid2007.140190. 24964003 PMC4073879

[57] Cheung AK, Ng TF, Lager KM, Alt DP, Delwart EL, Pogranichniy RM 2014 Unique circovirus-like genome detected in pig feces. Genome Announc 2:e00251-14. doi:10.1128/genomeA.00251-14. 24723710 PMC3983299

[58] Woo PCY, Lau SKP, Teng JLL, Tsang AKL, Joseph M, Wong EYM, Tang Y, Sivakumar S, Bai R, Wernery R, Wernery U, Yuen K-Y 2014 Metagenomic analysis of viromes of dromedary camel fecal samples reveals large number and high diversity of circoviruses and picobirnaviruses. Virology 471–473:117–125. doi:10.1016/j.virol.2014.09.020. PMC7112128 25461537

[59] Kraberger S, Argüello-Astorga GR, Greenfield LG, Galilee C, Law D, Martin DP, Varsani A 2015 Characterisation of a diverse range of circular replication-associated protein encoding DNA viruses recovered from a sewage treatment oxidation pond. Infect Genet Evol 31:73–86. doi:10.1016/j.meegid.2015.01.001. 25583447

[60] Roux S, Enault F, Hurwitz BL, Sullivan MB 2015 VirSorter: mining viral signal from microbial genomic data. PeerJ 3:e985. doi:10.7717/peerj.985. 26038737 PMC4451026

[61] Buchfink B, Xie C, Huson DH 2015 Fast and sensitive protein alignment using DIAMOND. Nat Methods 12:59–60. doi:10.1038/nmeth.3176. 25402007

[62] Bolduc B, Jang HB, Doulcier G, You Z-Q, Roux S, Sullivan MB 2017 vConTACT: an iVirus tool to classify double-stranded DNA viruses that infect *Archaea* and *Bacteria*. PeerJ 5:e3243. doi:10.7717/peerj.3243. 28480138 PMC5419219

[63] Yinda CK, Zeller M, Conceicaõ-Neto N, Maes P, Deboutte W, Beller L, Heylen E, Ghogomu SM, Van Ranst M, Matthijnssens J 2016 Novel highly divergent reassortant bat rotaviruses in Cameroon, without evidence of zoonosis. Sci Rep 6:34209. doi:10.1038/srep34209. 27666390 PMC5035928

[64] Clokie MRJ, Millard AD, Letarov AV, Heaphy S 2011 Phages in nature. Bacteriophage 1:31–45. doi:10.4161/bact.1.1.14942. 21687533 PMC3109452

[65] Ayukekbong JA, Fobisong C, Lindh M, Nkuo-Akenji T, Bergström T, Norder H 2014 Molecular analysis of enterovirus in Cameroon by partial 5′UTR-VP4 gene sequencing reveals a high genetic diversity and frequency of infections. J Med Virol 86:2092–2101. doi:10.1002/jmv.23926. 24634190

[66] Sadeuh-Mba SA, Bessaud M, Joffret M-L, Endegue Zanga M-C, Balanant J, Mpoudi Ngole E, Njouom R, Reynes J-M, Delpeyroux F, Rousset D 2014 Characterization of enteroviruses from non-human primates in Cameroon revealed virus types widespread in humans along with candidate new types and species. PLoS Negl Trop Dis 8:e3052. doi:10.1371/journal.pntd.0003052. 25079078 PMC4117447

[67] Cascio A, Bosco M, Vizzi E, Giammanco A, Ferraro D, Arista S 1996 Identification of picobirnavirus from faeces of Italian children suffering from acute diarrhea. Eur J Epidemiol 12:545–547. 8905320 10.1007/BF00144011 PMC7088005

[68] Pereira H, Linhares A, Candeias JA, Glass R 1993 National laboratory surveillance of viral agents of gastroenteritis in Brazil. Pan Am J Public Heal 27:224–233. 8220517

[69] Lim ES, Wang D, Holtz LR 2016 The bacterial microbiome and virome milestones of infant development. Trends Microbiol 24:801–810. doi:10.1016/j.tim.2016.06.001. 27353648

[70] Lim ES, Zhou Y, Zhao G, Bauer IK, Droit L, Ndao IM, Warner BB, Tarr PI, Wang D, Holtz LR 2015 Early life dynamics of the human gut virome and bacterial microbiome in infants. Nat Med 21:1228–1234. doi:10.1038/nm.3950. 26366711 PMC4710368

[71] Hong Y, Dover SL, Cole TE, Brasier CM, Buck KW 1999 Multiple mitochondrial viruses in an isolate of the Dutch elm disease fungus Ophiostoma novo-ulmi. Virology 258:118–127. doi:10.1006/viro.1999.9691. 10329574

[72] Krishnamurthy SR, Wang D 2018 Extensive conservation of prokaryotic ribosomal binding sites in known and novel picobirnaviruses. Virology 516:108–114. doi:10.1016/j.virol.2018.01.006. 29346073

[73] Dyall SD, Brown MT, Johnson PJ 2004 Ancient invasions: from endosymbionts to organelles. Science 304:253–257. doi:10.1126/science.1094884. 15073369

[74] Simon AK, Hollander GA, McMichael A 2015 Evolution of the immune system in humans from infancy to old age. Proc R Soc B Biol Sci 282: 20143085. doi:10.1098/rspb.2014.3085. PMC4707740 26702035

[75] Katz JM, Plowden J, Renshaw-Hoelscher M, Lu X, Tumpey TM, Sambhara S 2004 Immunity to influenza. Immunol Res 29:113–124. doi:10.1385/IR:29:1-3:113. 15181275

[76] Brodin P, Davis MM 2017 Human immune system variation. Nat Rev Immunol 17:21–29. doi:10.1038/nri.2016.125. 27916977 PMC5328245

[77] De Vlaminck I, Khush KK, Strehl C, Kohli B, Luikart H, Neff NF, Okamoto J, Snyder TM, Cornfield DN, Nicolls MR, Weill D, Bernstein D, Valantine HA, Quake SR 2013 Temporal response of the human virome to immunosuppression and antiviral therapy. Cell 155:1178–1187. doi:10.1016/j.cell.2013.10.034. 24267896 PMC4098717

[78] Béland K, Dore-Nguyen M, Gagné M-J, Patey N, Brassard J, Alvarez F, Halac U 2014 Torque Teno virus in children who underwent orthotopic liver transplantation: new insights about a common pathogen. J Infect Dis 209:247–254. doi:10.1093/infdis/jit423. 23922368

[79] Maggi F, Pifferi M, Fornai C, Andreoli E, Tempestini E, Vatteroni M, Presciuttini S, Marchi S, Pietrobelli A, Boner A, Pistello M, Bendinelli M 2003 TT virus in the nasal secretions of children with acute respiratory diseases: relations to viremia and disease severity. J Virol 77:2418–2425. 12551979 10.1128/JVI.77.4.2418-2425.2003 PMC141071

[80] Chung J-Y, Han TH, Koo JW, Kim SW, Seo JK, Hwang ES 2007 Small anellovirus infections in Korean children. Emerg Infect Dis 13:791–793. doi:10.3201/eid1305.061149. 18044047 PMC2738455

[81] Miyamoto M, Takahashi H, Sakata I, Adachi Y 2000 Hepatitis-associated aplastic anemia and transfusion-transmitted virus infection. Intern Med 39:1068–1070. 11197792 10.2169/internalmedicine.39.1068

[82] Spandole S, Cimponeriu D, Berca LM, Mihăescu G 2015 Human anelloviruses: an update of molecular, epidemiological and clinical aspects. Arch Virol 160:893–908. doi:10.1007/s00705-015-2363-9. 25680568

[83] Minot S, Sinha R, Chen J, Li H, Keilbaugh SA, Wu GD, Lewis JD, Bushman FD 2011 The human gut virome: inter-individual variation and dynamic response to diet. Genome Res 21:1616–1625. doi:10.1101/gr.122705.111. 21880779 PMC3202279

[84] Reyes A, Haynes M, Hanson N, Angly FE, Heath AC, Rohwer F, Gordon JI 2010 Viruses in the fecal microbiota of monozygotic twins and their mothers. Nature 466:334–338. doi:10.1038/nature09199. 20631792 PMC2919852

[85] Rohwer F 2003 Global phage diversity. Cell 113:141. 12705861 10.1016/s0092-8674(03)00276-9

[86] Conceicao-Neto N, Zeller M, Lefrere H, De Bruyn P, Beller L, Deboutte W, Yinda CK, Lavigne R, Maes P, Van Ranst M, Heylen E, Matthijnssens J 2015 Modular approach to customise sample preparation procedures for viral metagenomics: a reproducible protocol for virome analysis. Sci Rep 5:16532. doi:10.1038/srep16532. 26559140 PMC4642273

[87] Bolger AM, Lohse M, Usadel B 2014 Trimmomatic: a flexible trimmer for Illumina sequence data. Bioinformatics 30:2114–2120. doi:10.1093/bioinformatics/btu170. 24695404 PMC4103590

[88] Bankevich A, Nurk S, Antipov D, Gurevich AA, Dvorkin M, Kulikov AS, Lesin VM, Nikolenko SI, Pham S, Prjibelski AD, Pyshkin AV, Sirotkin AV, Vyahhi N, Tesler G, Alekseyev MA, Pevzner PA 2012 SPAdes: a new genome assembly algorithm and its applications to single-cell sequencing. J Comput Biol 19:455–477. doi:10.1089/cmb.2012.0021. 22506599 PMC3342519

[89] Marchler-Bauer A, Derbyshire MK, Gonzales NR, Lu S, Chitsaz F, Geer LY, Geer RC, He J, Gwadz M, Hurwitz DI, Lanczycki CJ, Lu F, Marchler GH, Song JS, Thanki N, Wang Z, Yamashita RA, Zhang D, Zheng C, Bryant SH 2015 CDD: NCBI’s conserved domain database. Nucleic Acids Res 43:D222–D226. doi:10.1093/nar/gku1221. 25414356 PMC4383992

[90] Kumar S, Stecher G, Tamura K 2016 MEGA7: molecular evolutionary genetics analysis version 7.0 for bigger datasets. Mol Biol Evol 33:1870–1874. doi:10.1093/molbev/msw054. 27004904 PMC8210823

[91] Katoh K, Misawa K, Kuma K, Miyata T 2002 MAFFT: a novel method for rapid multiple sequence alignment based on fast Fourier transform. Nucleic Acids Res 30:3059–3066. 12136088 10.1093/nar/gkf436 PMC135756

[92] Keane TM, Creevey CJ, Pentony MM, Naughton TJ, McInerney JO 2006 Assessment of methods for amino acid matrix selection and their use on empirical data shows that ad hoc assumptions for choice of matrix are not justified. BMC Evol Biol 6:29. doi:10.1186/1471-2148-6-29. 16563161 PMC1435933

[93] Stamatakis A 2014 RAxML version 8: a tool for phylogenetic analysis and post-analysis of large phylogenies. Bioinformatics 30:1312–1313. doi:10.1093/bioinformatics/btu033. 24451623 PMC3998144

[94] Bolduc B, Roux S 2017 Clustering viral genomes in iVirus. https://www.protocols.io/view/clustering-viral-genomes-in-ivirus-gwebxbe.

[95] Langmead B, Salzberg SL 2012 Fast gapped-read alignment with Bowtie 2. Nat Methods 9:357–359. doi:10.1038/nmeth.1923. 22388286 PMC3322381

[96] R Core Team. 2016 R: a language and environment for statistical computing. R Foundation for Statistical Computing, Vienna, Austria.

[97] Oksanen J, Blanchet FG, Kindt R, Legendre P, Minchin PR, O’Hara RB, Simpson GL, Solymos P, Stevens MHH, Wagner H 2017 vegan: Community Ecology Package https://cran.r-project.org/web/packages/vegan/index.html.

[98] Grissa I, Vergnaud G, Pourcel C 2007 The CRISPRdb database and tools to display CRISPRs and to generate dictionaries of spacers and repeats. BMC Bioinformatics 8:172. doi:10.1186/1471-2105-8-172. 17521438 PMC1892036

[99] Hyatt D, Chen G-L, LoCascio PF, Land ML, Larimer FW, Hauser LJ 2010 Prodigal: prokaryotic gene recognition and translation initiation site identification. BMC Bioinformatics 11:119. doi:10.1186/1471-2105-11-119. 20211023 PMC2848648

[100] Cline MS, Smoot M, Cerami E, Kuchinsky A, Landys N, Workman C, Christmas R, Avila-Campilo I, Creech M, Gross B, Hanspers K, Isserlin R, Kelley R, Killcoyne S, Lotia S, Maere S, Morris J, Ono K, Pavlovic V, Pico AR, Vailaya A, Wang P-L, Adler A, Conklin BR, Hood L, Kuiper M, Sander C, Schmulevich I, Schwikowski B, Warner GJ, Ideker T, Bader GD 2007 Integration of biological networks and gene expression data using Cytoscape. Nat Protoc 2:2366–2382. doi:10.1038/nprot.2007.324. 17947979 PMC3685583

