# Supplementary material for: Gut Virome Analysis of Cameroonians Reveals High Diversity of Enteric Viruses, Including Potential Interspecies Transmitted Viruses
Source: mSphere. 2019 Jan 23;4(1):e00585-18. doi: 10.1128/mSphere.00585-18 (PMC6344602; doi:10.1128/mSphere.00585-18)
Supplement: TABLE S1 [file mSphere.00585-18-st001.pdf]

Supplemental Table S1: Metadata pools and reads

Pool Number	Age group (years)	Contact with bats*	Location	Reads after trimming	Virus reads (%)	Phage reads[#] (%)	Eukaryotic virus reads[##] (%)	Others reads (%)
HP01	0-2.9	I	Kumba	6821225	1845731 (27.1)	1837808 (99.6)	7923 (0.4)	4975494 (72.9)
HP02	0-2.9	I	Lysoka	8659618	3383520 (39.1)	3309931 (97.8)	73589 (2.2)	5276098 (60.9)
HP03	3-19	I	Lysoka	5783225	5015710 (86.7)	112782 (2.2)	4902928 (97.8)	767515 (13.3)
HP04	3-19	I	Lysoka	5882399	440446 (7.5)	434693 (98.7)	5753 (1.3)	5441953 (92.5)
HP05	0-2.9	I	Lysoka	5505387	917476 (16.7)	899273 (98)	18203 (2)	4587911 (83.3)
HP06	3-19	D	Lysoka	7902091	808918 (10.2)	730485 (90.3)	78433 (9.7)	7093173 (89.8)
HP07	20-59	D	Kumba	8078419	1898171 (23.5)	1838758 (96.9)	59413 (3.1)	6180248 (76.5)
HP08	3-19	D	Lysoka	6372426	813054 (12.8)	805901 (99.1)	7153 (0.9)	5559372 (87.2)
HP09	3-19	D	Lysoka	15488238	973410 (6.3)	961560 (98.8)	11850 (1.2)	14514828 (93.7)
HP10	3-19	D	Lysoka	30267100	2741917 (9.1)	2424683 (88.4)	317234 (11.6)	27525183 (90.9)
HP11	0-2.9	N	Kumba	6424025	1592461 (24.8)	1532208 (96.2)	60253 (3.8)	4831564 (75.2)
HP12	0-2.9	N	Kumba	8938045	2289364 (25.6)	2266613 (99)	22751 (1)	6648681 (74.4)
HP13	0-2.9	N	Kumba	7172273	759472 (10.6)	759450 (100)	22 (0)	6412801 (89.4)
HP14	0-2.9	N	Kumba	8317276	2904795 (34..9)	2874272 (98.9)	30523 (1.1)	5412481 (65.1)
HP15	0-2.9	N	Kumba	6496464	1809123 (27.8)	1805705 (99.8)	3418 (0.2)	4687341 (72.2)
HP16	0-2.9	N	Kumba	5735230	1836646 (32)	1824983 (99.4)	11663 (0.6)	3898584 (68)
HP17	0-2.9	N	Kumba	6531053	3302164 (50.6)	3302126 (100)	38 (0)	3228889 (49.4)
HP18	3-19	N	Lysoka	7386100	3147511 (42.6)	3134085 (99.6)	13426 (0.4)	4238589 (57.4)
HP19	20-59	D	Kumba	6497261	312500 (4.8)	312486 (100)	14 (0)	6184761 (95.2)
HP20	20-59	D	Kumba	6541190	1086905 (16.6)	1083357 (99.7)	3548 (0.3)	5454285 (83.4)
HP21	60+	D	Kumba	6226076	3280492 (52.7)	3275248 (99.8)	5244 (0.2)	2945584 (47.3)
HP22	60+	D	Kumba	8659047	2155163 (24.9)	2152470 (99.9)	2693 (0.1)	6503884 (75.1)
HP23	20-59	D	Kumba	7131387	2297334 (32.2)	2295926 (99.9)	1408 (0.1)	4834053 (67.8)
HP24	20-59	D	Kumba	7798630	1641048 (21)	1634668 (99.6)	6380 (0.4)	6157582 (79)
HP25	60+	D	Kumba	5497780	2438494 (44.4)	2436703 (99.9)	1791 (0.1)	3059286 (55.6)
HP26	20-59	D	Lysoka	5771755	972015 (16.8)	970032 (99.8)	1983 (0.2)	4799740 (83.2)
HP27	20-59	D	Lysoka	6102352	804604 (13.2)	804336 (100)	268 (0)	5297748 (86.8)

HP28	20-59	D	Lysoka	6157697	890287 (14.5)	882363 (99.1)	7924 (0.9)	5267410 (85.5)
HP29	20-59	D	Kumba	8723328	1443720 (16.6)	1440186 (99.8)	3534 (0.2)	7279608 (83.4)
HP30	20-59	D	Kumba	5120977	2048543 (40)	2048440 (100)	103 (0)	3072434 (60)
HP31	20-59	D	Kumba	6020103	1440227 (23.9)	1439366 (99.9)	861 (0.1)	4579876 (76.1)
HP32	20-59	D	Kumba	5596334	834043 (14.9)	828993 (99.4)	5050 (0.6)	4762291 (85.1)
HP33	20-59	D	Kumba	6086553	597899 (9.8)	597893 (100)	6 (0)	5488654 (90.2)
HP34	20-59	D	Kumba	6367060	3258637 (51.2)	3255679 (99.9)	2958 (0.1)	3108423 (48.8)
HP35	3-19	I	Lysoka	11629199	975804 (8.4)	567450 (58.2)	408354 (41.8)	10653395 (91.6)
HP36	3-19	I	Lysoka	10877814	2576296 (23.7)	2575283 (100)	1013 (0)	8301518 (76.3)
HP37	3-19	I	Lysoka	11875672	2664120 (22.4)	2655829 (99.7)	8291 (0.3)	9211552 (77.6)
HP38	3-19	I	Lysoka	10477247	768030 (7.3)	741315 (96.5)	26715 (3.5)	9709217 (92.7)
HP39	3-19	I	Lysoka	11714844	2621778 (22.4)	2608220 (99.5)	13558 (0.5)	9093066 (77.6)
HP40	3-19	I	Lysoka	9214626	1280734 (13.9)	1279119 (99.9)	1615 (0.1)	7933892 (86.1)
HP41	3-19	I	Lysoka	11716365	452643 (3.9)	448978 (99.2)	3665 (0.8)	11263722 (96.1)
HP42	3-19	I	Lysoka	10665321	1735994 (16.3)	1731616 (99.7)	4378 (0.3)	8929327 (83.7)
HP43	20-59	I	Lysoka	7677258	167297 (2.2)	165218 (98.8)	2079 (1.2)	7509961 (97.8)
HP44	20-59	I	Kumba	13149162	1716814 (13.1)	1598456 (93.1)	118358 (6.9)	11432348 (86.9)
HP45	0-2.9	N	Kumba	7075115	5266256 (74.4)	208553 (4)	5057703 (96)	1808859 (25.6)
HP46	0-2.9	N	Kumba	4898398	2695244 (55)	394419 (14.6)	2300825 (85.4)	2203154 (45)
HP47	0-2.9	I	Kumba	4679565	782913 (16.7)	743603 (95)	39310 (5)	3896652 (83.3)
HP48	0-2.9	I	Lysoka	5107629	2998473 (58.7)	1845695 (61.6)	1152778 (38.4)	2109156 (41.3)
HP49	0-2.9	N	Kumba	4430633	1156664 (26.1)	1119802 (96.8)	36862 (3.2)	3273969 (73.9)
HP50	0-2.9	N	Kumba	4412948	362260 (8.2)	362218 (100)	42 (0)	4050688 (91.8)
HP51	0-2.9	N	Kumba	6614502	1513991 (22.9)	1512785 (99.9)	1206 (0.1)	5100511 (77.1)
HP52	0-2.9	N	Kumba	6108719	1967777 (32.2)	1953321 (99.3)	14456 (0.7)	4140942 (67.8)
HP53	0-2.9	N	Kumba	5739089	2825362 (49.2)	2806707 (99.3)	18655 (0.7)	2913727 (50.8)
HP54	0-2.9	N	Kumba	3175327	1769023 (55.7)	1769023 (100)	0 (0)	1406304 (44.3)
HP55	0-2.9	N	Kumba	5624938	4958824 (88.2)	611293 (12.3)	4347531 (87.7)	666114 (11.8)
HP56	0-2.9	I	Kumba	3971571	330698 (8.3)	329998 (99.8)	700 (0.2)	3640873 (91.7)
HP57	3-19	N	Kumba	6907327	1487259 (21.5)	1476341 (99.3)	10918 (0.7)	5420068 (78.5)

HP58	3-19	N	Kumba	5231886	2930395 (56)	2922399 (99.7)	7996 (0.3)	2301491 (44)
HP59	20-59	I	Kumba	5646203	2074259 (36.7)	2067634 (99.7)	6625 (0.3)	3571944 (63.3)
HP60	20-59	N	Kumba	4452181	990540 (22.2)	986827 (99.6)	3713 (0.4)	3461641 (77.8)
HP61	20-59	N	Kumba	4856427	1351722 (27.8)	1350447 (99.9)	1275 (0.1)	3504705 (72.2)
HP62	20-59	I	Lysoka	4744174	765658 (16.1)	765631 (100)	27 (0)	3978516 (83.9)
HP63	60+	D	Kumba	6146243	860975 (14)	587449 (68.2)	273526 (31.8)	5285268 (86)

* D: direct contact with bats; I: indirect contact with bats; N: no contact with bats
# Determined using VirSorter
Determined using DIAMOND
