# Supplementary figures and images for: Gut Virome Analysis of Cameroonians Reveals High Diversity of Enteric Viruses, Including Potential Interspecies Transmitted Viruses
Source: mSphere. 2019 Jan 23;4(1):e00585-18. doi: 10.1128/mSphere.00585-18 (PMC6344602; doi:10.1128/mSphere.00585-18)

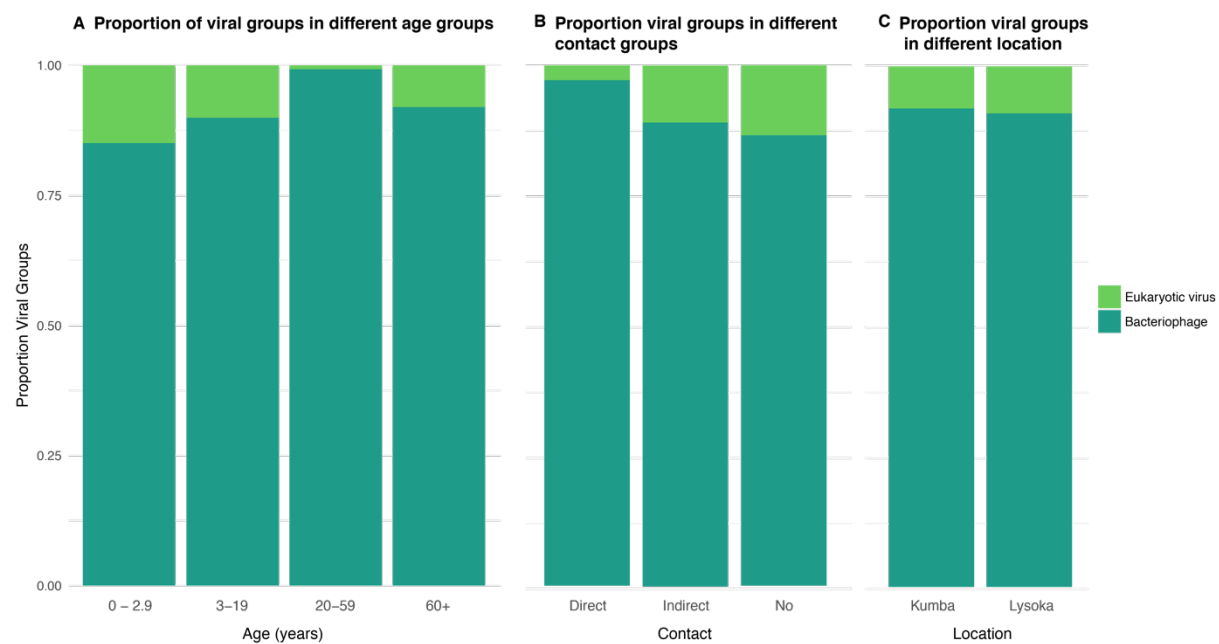

Fig. S1

Supplement: FIG S1 [file mSphere.00585-18-sf001.pdf]

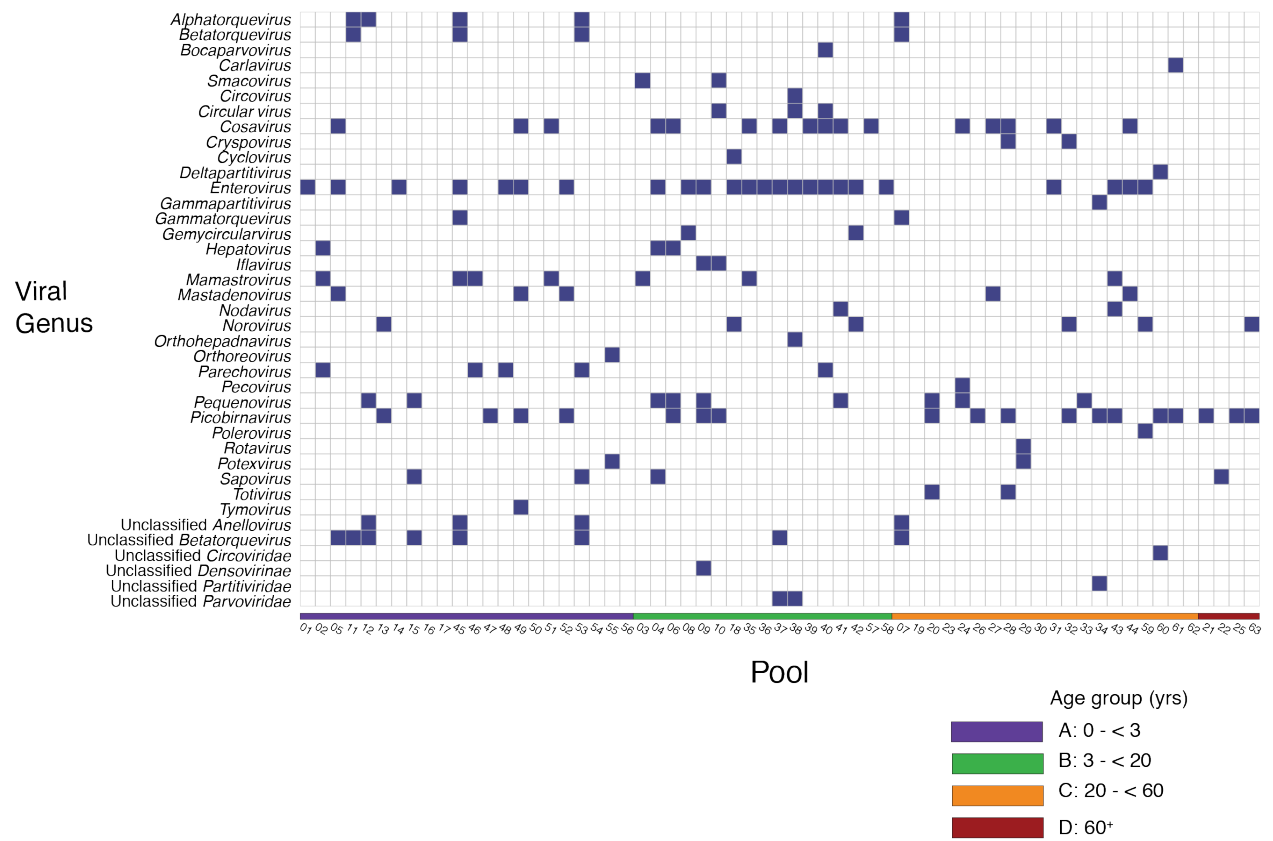

Fig. S2

Supplement: FIG S2 [file mSphere.00585-18-sf002.pdf]

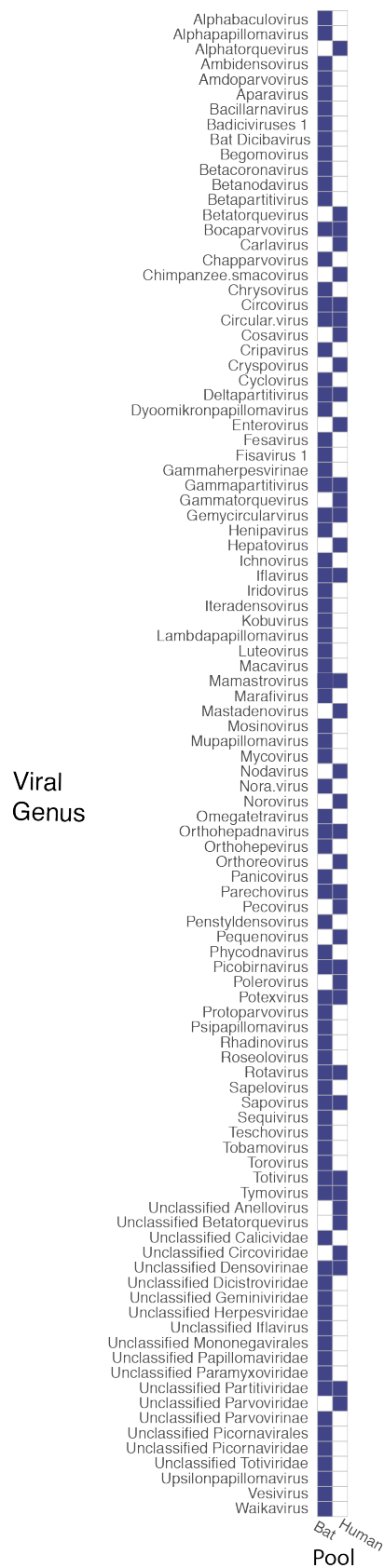

Fig. S3

Supplement: FIG S3 [file mSphere.00585-18-sf003.pdf]

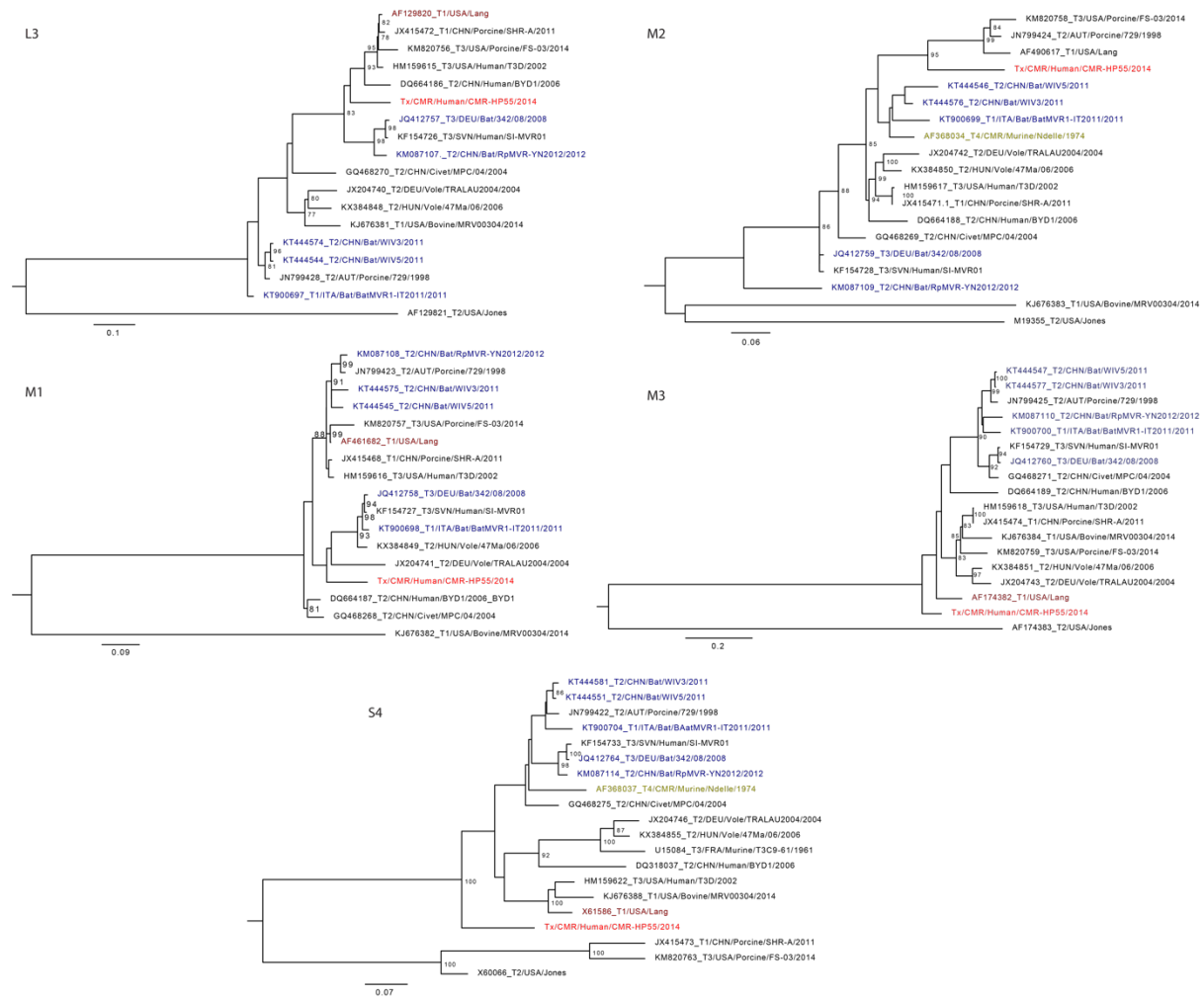

Fig. S4

Supplement: FIG S4 [file mSphere.00585-18-sf004.pdf]

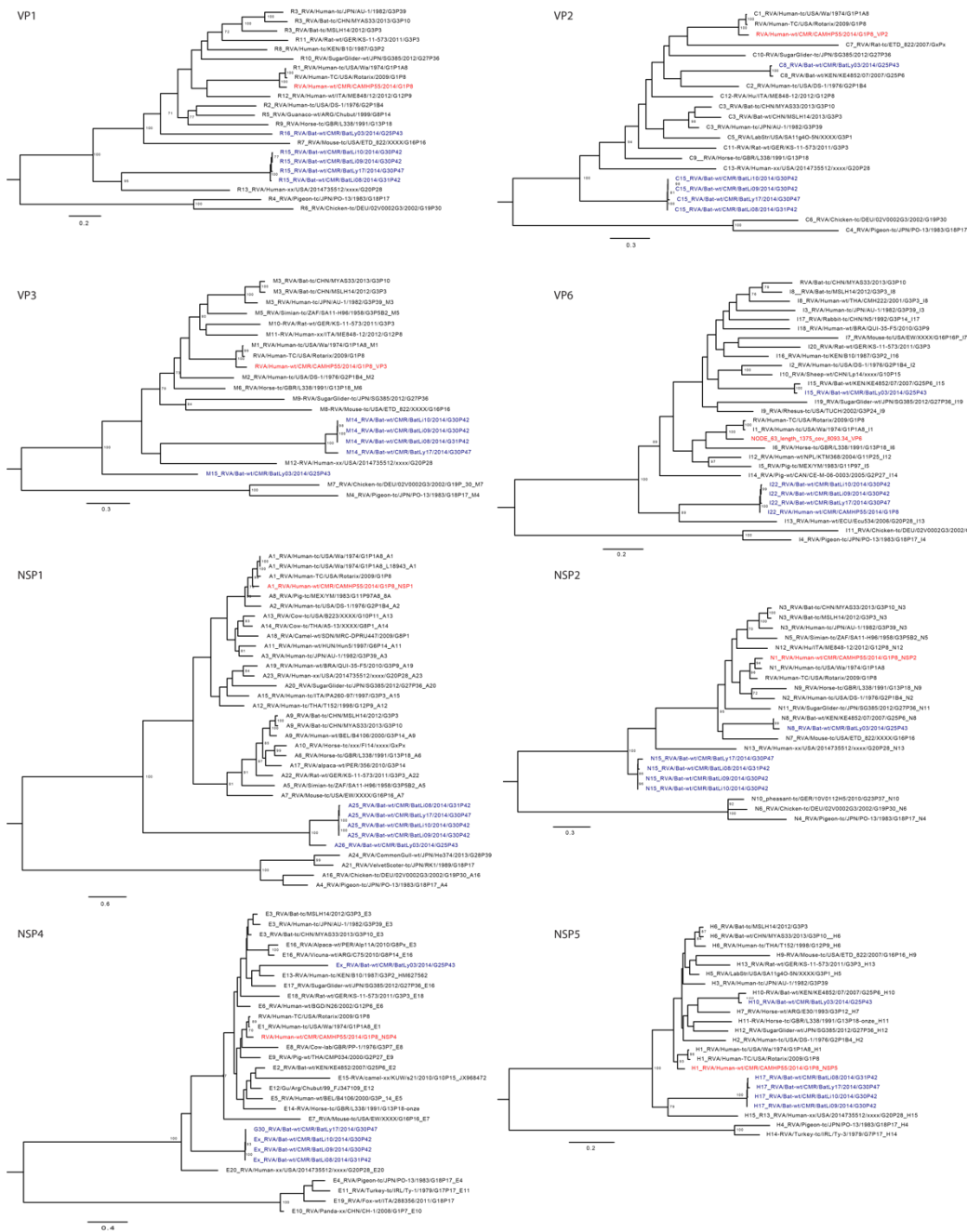

Fig. S5

Supplement: FIG S5 [file mSphere.00585-18-sf005.pdf]

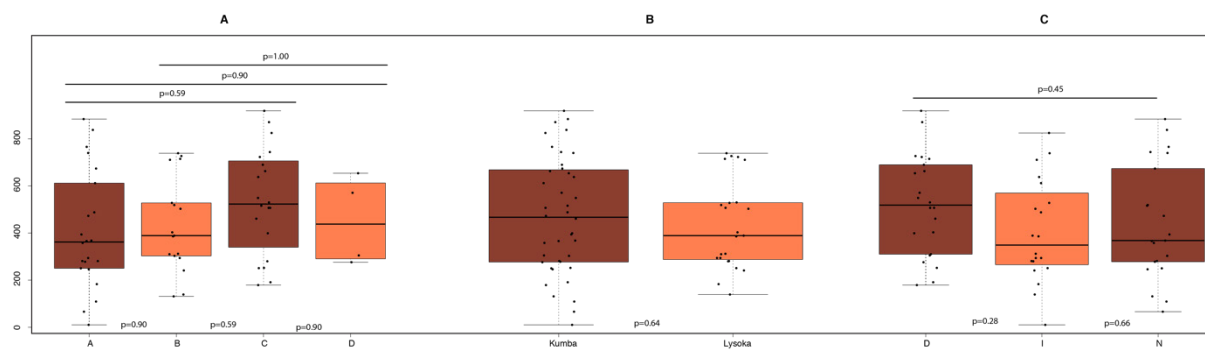

Fig. S6

Supplement: FIG S6 [file mSphere.00585-18-sf006.pdf]
