# Supplementary material for: Gut Virome Analysis of Cameroonians Reveals High Diversity of Enteric Viruses, Including Potential Interspecies Transmitted Viruses
Source: mSphere. 2019 Jan 23;4(1):e00585-18. doi: 10.1128/mSphere.00585-18 (PMC6344602; doi:10.1128/mSphere.00585-18)
Supplement: TABLE S2 [file mSphere.00585-18-st002.pdf]

Supplemental Table S2: Results of CRISPR sequences BLASTn against the phage community revealing a diverse bacterial community.

Pool	°Contig size	Spacer CRISPR sequence	*E-value	#Accession no.	Bacteria species	Family
HP1	1486	GCTGATTCCACGTGTCCGGCGTGCCGAAAATGGC	2.70e-11	NZ_CP013673	Bifidobacterium longum strain 35624	Bifidobacteriaceae
HP7	5734	CCAGCGGGAATGTGCCGGGTTCAACTGGACGC	5.01e-08	NZ_CP007731	Klebsiella pneumoniae subsp. pneumoniae KPNIH27	Enterobacteriaceae
HP12	15125	CGCACTAAGACGCTCAGTCCAGGCTTTGATCTG	9.01e-11	NC_012731	Klebsiella pneumoniae subsp. pneumoniae NTUH-K2044	Enterobacteriaceae
HP12	15125	ATTGCCTGACGCCAGGCTATTTCGATATCTGAG	9.01e-11	NC_012731	Klebsiella pneumoniae subsp. pneumoniae NTUH-K2044	Enterobacteriaceae
HP12	5942	CTTCTGCTGATACTCCGCCTTAATCGCTTTGC	1.08e-09	NC_013282	Cronobacter turicensis z3032	Enterobacteriaceae
HP12	15125	GCTAACCAGTGGATAGAGCACTATGTGACGAC	1.39e-08	NZ_CP008929	Klebsiella pneumoniae strain PMK1	Enterobacteriaceae
HP12	21532	AGAATATTCAACTCCAGCGGGAAAAAGACGCA	2.99e-10	NZ_CP016406	Salmonella enterica subsp. enterica serovar Infantis strain FSIS1502169	Enterobacteriaceae
HP14	2989	CAATTGCGTTTCCCTGTATCTCGGTTGTGTCAACC G	2.38e-12	NC_015160	Odoribacter splanchnicus DSM 20714	Odoribacteraceae
HP16	41227	CCTGTGGTCGGACAGGTGACGCCAGCAGAT	1.16e-08	NZ_CP008816	Enterococcus faecalis ATCC 29212	Enterococcaceae
HP18	39305	GCCTAAAGTTAGATATTTACCCTCAGACTTTC	2.99e-10	NC_015760	Odoribacter splanchnicus DSM 20715	Odoribacteraceae
HP18	4042	TTCTTGGTCTTAACTTCAAAGAGGCTAATTCTAT	2.70e-11	NC_021044	Eubacterium rectale M104/1	Eubacteriaceae
HP18	7827	GTTTCCTATTGTAAGTACATACGAATCATA	3.23e-09	NZ_LN877293	Bacteroides fragilis genome assembly BFBE1.1	Bacteroidaceae
HP23	7550	GTGCTCAGGTTGAGCTGCACGGTGTTCGGGTGG	4.19e-09	NZ_CP014010	Klebsiella pneumoniae subsp. pneumoniae strain RJF999	Enterobacteriaceae
HP24	6595	TGACCAGTAATCACATAATCCAAAAGCGGAACAA CG	8.58e-12	NC_015160	Odoribacter splanchnicus DSM 20712	Odoribacteraceae
HP24	6595	GAAATATTGTTATTTTGTTAGTCTCACTTATAAAA A	2.38e-12	NC_015160	Odoribacter splanchnicus DSM 20713	Odoribacteraceae
HP24	1339	TGTTGCAAACATGAGAAACGGCATGGGAGTATAT	2.70e-11	NC_015977	Roseburia hominis A2-183	Lachnospiraceae
HP24	41462	ATTGGATTACACCTAAAAGCATCAATCAATGAG	9.01e-11	NC_015977	Roseburia hominis A2-184	Lachnospiraceae
HP26	40621	CTAATCATCCATTTCGTCTATGATGTAGGCTGTTTT	5.16e-09	NC_015873	Megasphaera elsdenii strain DSM 20460	Veillonellaceae
HP26	16367	TGACCGTTTCGATCACCCACGGCGGCTTCTCC	1.08e-09	NZ_AP012322	Bifidobacterium angulatum DSM 20098	Bifidobacteriaceae
HP26	16367	CAAGTGGGCGCAGGCATTGGCCGCGCACCGCAAT GA	2.38e-12	NZ_CP007456	Bifidobacterium kashiwanohense PV20-2	Bifidobacteriaceae
HP27	40621	TTCTTGTTCAAGCTTTAACTGTTCTTGCATCGTTCC CT	2.68e-12	NC_015873	Megasphaera elsdenii strain DSM 20461	Veillonellaceae

HP29	94125	CTATTGATGAGGTGCACCATCAGAAGCGAGAT	2.99e-10	NC_009800	Escherichia coli HS	Enterobacteriaceae
HP29	94125	CTGGATTTCACCTCAGCAAATGCTGGATGTGG	2.99e-10	NC_011751	Escherichia coli UMN026	Enterobacteriaceae
HP30	55511	AAAAATGTTTCTGATTGCAAAAGCAGATTA	3.23e-09	NC_012781	[Eubacterium] rectale ATCC 33656	Lachnospiraceae
HP31	23662	ACTGTTTCAGCAACAATGCTAATCAAATC	1.16e-08	NC_016826	Streptococcus infantarius subsp. infantarius CJ18	Streptococcaceae
HP31	2282	TATAATCACCTCCTTTCGAGATGATTATAT	1.16e-08	NZ_CP014699	Streptococcus pantholopis strain TA	Streptococcaceae
HP32	1609	TACGAGCTCCCAACATACGTTGACGGTGCA	3.23e-09	NC_018221	Enterococcus faecalis D32	Enterococcaceae
HP32	2164	ACTGATATCACCGAACTGGCGCAGAGCCTGGA	3.87e-09	NZ_AP014950	Klebsiella pneumoniaeÊ	Enterobacteriaceae
HP32	2099	AAGAGGAAAAGGAATTTCGCCACTGTATATGG	2.99e-10	NZ_CP007222	Salmonella enterica subsp. enterica serovar Montevideo	Enterobacteriaceae
HP36	45545	CCAGCGGGAATGTGCCGGGTTCAACTGGACGC	5.01e-08	NZ_CP007731	Klebsiella pneumoniae subsp. pneumoniae KPNIH28	Enterobacteriaceae
HP46	3016	CGCAGACCCTCGGCGACTTCTACGTCAAGTCCCT	8.04e-12	NC_020517	Bifidobacterium breve UCC2003	Bifidobacteriaceae
HP46	3016	GAGTGTTTCCGCGTCCTGAAAGAGCATGGCGT	2.99e-10	NZ_AP012322	Bifidobacterium angulatum DSM 20098	Bifidobacteriaceae
HP47	22358	CGCTCTTCATTGTTTAACTCTAAATGCAAT	3.23e-09	NC_013798	Streptococcus gallolyticus UCN34	Streptococcaceae
HP47	22358	GCGTTTTAAGCTCTTGCATATAATCTTCTG	3.23e-09	NC_017576	Streptococcus gallolyticus subsp. gallolyticus ATCC 43143	Streptococcaceae
HP48	7406	ATTCGTGAAATGACGGGTGCAGATGAAGAAAGTT TACAAGTGA	4.21e-16	NC_013520	Veillonella parvula DSM 2008	Veillonellaceae
HP48	7406	CAGAGAACGTTGATATGACTACACAAGCTATCAG CGG	1.18e-10	NC_013520	Veillonella parvula DSM 2008	Veillonellaceae
HP48	40428	TGGAGGATCCGGAAAACACGCGCAAGCTCAAGCG CT	2.38e-12	NC_014624	Eubacterium limosum KIST612	Eubacteriaceae
HP48	40428	GCTAAAGGATTGGATTGATATCTTCCTTGGCATCT GG	7.04e-13	NC_014624	Eubacterium limosum KIST613	Eubacteriaceae
HP48	40428	GGACAGCTTTACCCGCAAGGCCTATGCCCTTTACG A	2.38e-12	NC_014624	Eubacterium limosum KIST614	Eubacteriaceae
HP48	6842	CGCAGACCCTCGGCGACTTCTACGTCAAGTCCCT	1.62e-08	NC_020517	Bifidobacterium breve UCC2003	Bifidobacteriaceae
HP48	6842	CATGCAGCATGATCTCCGCGAACGCGATCACCGC	3.49e-10	NZ_CP007456	Bifidobacterium kashiwanohense PV20-2	Bifidobacteriaceae
HP48	40428	TTGGCTTTCTGGTTGATGGCCGTCGCGGTTTCGGA	8.04e-12	NZ_CP011914	Eubacterium limosum strain SA11	Eubacteriaceae
HP48	25252	TGTTGCCATTTGAATGCCTCCTAAATATTT	1.16e-08	NZ_CP014881	Lactobacillus backii strain TMW 1.1991	Lactobacillaceae
HP51	3505	CGGCGCGCCAGGCGTTATTGTCATCCACCCGCA	9.01e-11	NC_012731	Klebsiella pneumoniae subsp. pneumoniae NTUH-K2044	Enterobacteriaceae
HP51	3505	AAAGTAGAGCGTTAAATAACACACTGTAATCA	2.99e-10	NC_013850	Klebsiella variicola At-23	Enterobacteriaceae
HP51	3505	GCGGGTGGATGACAATAACGCCTGGCGCGCCG	2.99e-10	NZ_CP016813	Klebsiella pneumoniae strain ED2	Enterobacteriaceae

HP52	46519	TGGCGCAAGTATGCAATTTGAAGCCTCGT	1.16e-08	NC_016776	Roseburia hominis A2-183	Lachnospiraceae
HP53	46519	GAAGCGGGCGAGGCTTTGACGTATGGAGGC	1.16e-08	NC_016776	Bacteroides fragilis 638R	Bacteroidaceae
HP53	19535	GCACATTCCAGGGCCAATTTCTTAATATCATA	3.87e-09	NC_017910	Shimwellia blattae DSM 4481	Enterobacteriaceae
HP53	14271	AAATGATGGTGATAAAACCATACTGAAACAGG	2.99e-10	NC_017910	Shimwellia blattae DSM 4481	Enterobacteriaceae
HP53	28142	AAAAAGTCCTCCTGGTGCTGTTATTTAGTA	3.23e-09	NZ_CP013476	Turicibacter sp. H121	Erysipelotrichaceae
HP53	28142	GAGAGTTTAAAATCCCTGGCAAGGCACAAG	3.23e-09	NZ_CP013476	Turicibacter sp. H124	Erysipelotrichaceae
HP53	28142	ATAACTCTTAAAGGGGGAACGGATGTGGCA	3.23e-09	NZ_CP013476	Turicibacter sp. H125	Erysipelotrichaceae
HP55	8292	ATCTTGCCTACGATTGACACCTCAACCGGTGA	2.99e-10	NC_010610	Lactobacillus fermentum IFO 3956	Lactobacillaceae
HP55	7375	TGCTGACAAGGTATTTAAGGACGCCGAGAAAC	2.99e-10	NC_010610	Lactobacillus fermentum IFO 3956	Lactobacillaceae
HP55	10377	CAGTTTTACAATGACTACGGGATCCATTGG	3.23e-09	NZ_CP011536	Lactobacillus fermentum 3872	Lactobacillaceae
HP55	8292	TCCCATTGATACTATACCAACCAACATCTGCTT	3.24e-10	NZ_CP011536	Lactobacillus fermentum 3872	Lactobacillaceae
HP57	21413	AGCCATATTTCCCAAATATCGGGCTGCACCAA	2.99e-10	NC_013850	Klebsiella variicola At-22	Enterobacteriaceae
HP57	21140	GGGATGTGGTTTACTGCGATCCGCCTTACGAA	2.99e-10	NC_013850	Klebsiella variicola At-24	Enterobacteriaceae
HP57	21413	GCGGCCGGAGTCAGACCGAATTCAACCAGGTA	2.99e-10	NZ_CP007222	Salmonella enterica subsp. enterica serovar Montevideo	Enterobacteriaceae
HP57	21413	TTGAGATGGCTCCAATGCTAGGTCATGGAATGG	9.01e-11	NZ_CP009208	Klebsiella pneumoniae subsp. pneumoniae strain ATCC 43816 KPPR1	Enterobacteriaceae
HP57	21140	TGTTGCCATTTGAATGCCTCCTAAATATTT	9.01e-11	NZ_CP015134	Klebsiella pneumoniae strain ATCC 35657	Enterobacteriaceae
HP57	21140	TCCCCTACCACCCACTGAGCAAACTGGTAGCA	2.99e-10	NZ_CP017934	Klebsiella pneumoniae strain CAV1016	Enterobacteriaceae
HP57	21140	CAGATACCAACGGTTACTACGGCAATCAGCAC	2.99e-10	NZ_CP017934	Klebsiella pneumoniae strain CAV1016	Enterobacteriaceae
HP58	3858	CTTTTTTATTTGATAAGGCTAAGAATGGGG	1.16e-08	NZ_CP013476	Turicibacter sp. H122	Erysipelotrichaceae
HP62	47608	ACAAAATAGACAAAATGATTTCAGAGATTGATA	1.17e-09	NZ_CP014010	Klebsiella pneumoniae subsp. pneumoniae strain RJF999	Enterobacteriaceae
HP63	5253	GAGAGTTTAAAATCCCTGGCAAGGCACAAG	3.23e-09	NZ_CP013476	Turicibacter sp. H123	Erysipelotrichaceae

°Contigs identified as phage by Virsorter
*E-value the CRISPR spacer and phage.
#Accession number of bacteria species corresponding to the CRISPR spacer.
