# Supplementary material for: Gut Virome Analysis of Cameroonians Reveals High Diversity of Enteric Viruses, Including Potential Interspecies Transmitted Viruses
Source: mSphere. 2019 Jan 23;4(1):e00585-18. doi: 10.1128/mSphere.00585-18 (PMC6344602; doi:10.1128/mSphere.00585-18)
Supplement: TABLE S3 [file mSphere.00585-18-st003.pdf]

Supplemental Table S3A: Accession numbers of all viruses described in the study.

Virus	Strain	Accession number
**Norovirus**	NV/CMRHP1/CMR/2014	MH608285
	NV/CMRHP18/CMR/2014	MH608286
	NV/CMRHP59/CMR/2014	MH608287
	Human-AstroV CMRHP2	MH933752
**Astrovirus**	Human-AstroV CMRHP3	MH933753
	Human-AstroV CMRHP6	MH933754
	Human-AstroV CMRHP45	MH933755
	Human-AstroV CMRHP46	MH933756
	Human-AstroV CMRHP35D	MH933757
	Human-AstroV CMRHP34	MH933758
	Human-AstroV CMRHP43	MH933759
**Cosavirus**	HCoSV/CMRHP6A/CMR/2014	MH933760
	HCoSV/CMRHP6B/CMR/2014	MH933761
	HCoSV/CMRHP24/CMR/2014	MH933762
	HCoSV/CMRHP44/CMR/2014	MH933763
	HCoSV/CMRHP49/CMR2014	MH933764
	HCoSV/CMRHP57/CMR/2014	MH933765
**Hepatitis A virus**	HAV/Human/CMRHP2/CMR/2014	MH933766
	HAV/Human/CMRHP4/CMR/2014	MH933767
	HAV/Human/CMRHP6/CMR/2014	MH933768
**Mammalian orthoreovirus**	T2/CMR/Human/CMR-HP55/2014_L1	MH933769
	T2/CMR/Human/CMR-HP55/2014_L2	MH933770
	T2/CMR/Human/CMR-HP55/2014_L3	MH933771
	T2/CMR/Human/CMR-HP55/2014_M1	MH933772
	T2/CMR/Human/CMR-HP55/2014_M2	MH933773
	T2/CMR/Human/CMR-HP55/2014_M3	MH933774
	T2/CMR/Human/CMR-HP55/2014_S1	MH933775
	T2/CMR/Human/CMR-HP55/2014_S2	MH933776
	T2/CMR/Human/CMR-HP55/2014_S3	MH933777
	T2/CMR/Human/CMR-HP55/2014_S4	MH933778
**Parechovirus**	HPeV-16/CMRHP2/CMR/2014	MH933779
	HPeV-1/CMRHP46/CMR/2014	MH933780
	HPeV-1/CMRHP48/CMR/2014	MH933781
**Rotavirus A**	RVA/Human-wt/CMR/CMRHP55/2014/G1P8_VP1	MH933782
	RVA/Human-wt/CMR/CMRHP55/2014/G1P8_VP2	MH933783
	RVA/Human-wt/CMR/CMRHP55/2014/G1P8_VP3	MH933784
	RVA/Human-wt/CMR/CMRHP55/2014/G1P8_VP4	MH933785
	RVA/Human-wt/CMR/CMRHP55/2014/G1P8_VP6	MH933786
	RVA/Human-wt/CMR/CMRHP55/2014/G1P8_VP7	MH933787
	RVA/Human-wt/CMR/CMRHP55/2014/G1P8_NSP1	MH933788
	RVA/Human-wt/CMR/CMRHP55/2014/G1P8_NSP2	MH933789
	RVA/Human-wt/CMR/CMRHP55/2014/G1P8_NSP4	MH933790
	RVA/Human-wt/CMR/CMRHP55/2014/G1P8_NSP5	MH933791

**Saffold virus**	CardioVirusB/CMRHP35/CMR/2014	MH933792
**Sapovirus**	HumanSaV/CMR/Lysoka-HP4/2014	MH933793
	HumanSaV/CMR/Kumba-HP22/2014	MH933794
	HumanSaV/CMR/Kumba-HP15/2014	MH933795
**Smacovirus**	HuSCV-CMRHP10	MH933796
	HuSCV-CMRHP03	MH933797
**Pecovirus**	Hu_PeCV_CMRHP60	MH933798
**Sewage-associated-like virus**	HP38A_Sewage-associated-like virus 1	MH933799
	HP38B_Sewage-associated-like virus 2	MH933800
**Picobirnavirus**	PBV/Human/CMRHP6A//CMR/2014	MH933801
	PBV/Human/CMRHP12/CMR/2014	MH933802
	PBV/Human/CMRHP20A/CMR/2014	MH933803
	PBV/Human/CMRHP22A//CMR/2014	MH933804
	PBV/Human/CMRHP22B/CMR/2014	MH933805
	PBV/Human/CMRHP25A/CMR/2014	MH933806
	PBV/Human/CMRHP25B/CMR/2014	MH933807
	PBV/Human/CMRHP26A/CMR/2014	MH933808
	PBV/Human/CMRHP26B/CMR/2014	MH933809
	PBV/Human/CMRHP26C/CMR/2014	MH933810
	PBV/Human/CMRHP26D/CMR/2014	MH933811
	PBV/Human/CMRHP28/CMR/2014	MH933812
	PBV/Human/CMRHP32A/CMR/2014	MH933813
	PBV/Human/CMRHP32B/CMR/2014	MH933814
	PBV/Human/CMRHP32C/CMR/2014	MH933815
	PBV/Human/CMRHP34A/CMR/2014	MH933816
	PBV/Human/CMRHP34B/CMR/2014	MH933817
	PBV/Human/CMRHP35/CMR/2014	MH933818
	PBV/Human/CMRHP47A/CMR/2014	MH933819
	PBV/Human/CMRHP47B/CMR/2014	MH933820
	PBV/Human/CMRHP49A/CMR/2014	MH933821
	PBV/Human/CMRHP52/CMR/2014	MH933822
	PBV/Human/CMRHP63A/CMR/2014	MH933823
	PBV/Human/CMRHP63B/CMR/2014	MH933824
	PBV/Human/CMRHP10A/CMR/2014	MH933825
	Lysoka picobirna-like virus CMRHP10B	MH933826
	PBV/Human/CMRHP20B/CMR/2014	MH933827
	PBV/Human/CMRHP20C/CMR/2014	MH933828
	Kumba picobirna-like virus CMRHP21A	MH933829
	PBV/Human/CMRHP21B/CMR/2014	MH933830
	PBV/Human/CMRHP21C/CMR/2014	MH933831
	PBV/Human/CMRHP24/CMR/2014	MH933832
	PBV/Human/CMRHP26E/CMR/2014	MH933833
	PBV/Human/CMRHP32D/CMR/2014	MH933834
	PBV/Human/CMRHP49B/CMR/2014	MH933835
	PBV/Human/CMRHP60A/CMR/2014	MH933836
	PBV/Human/CMRHP60B/CMR/2014	MH933837

	PBV/Human/CMRHP60C/CMR/2014	MH933838
	PBV/Human/CMRHP61/CMR/2014	MH933839
	PBV/Human/CMRHP6B/CMR/2014	MH933840
	PBV/Human/CMRHP9A/CMR/2014	MH933841
	Lysoka picobirna-like virus CMRHP9B	MH933842
**Enterovirus**	EV/Human/CMRHP1/CMR/2014	MH933843
	EV/Human/CMRPHP3/CMR/2014	MH933844
	EV/Human/CMRHP5A/CMR/2014	MH933845
	EV/Human/CMRHP5B/CMR/2014	MH933846
	EV/Human/CMRHP8A/CMR/2014	MH933847
	EV/Human/CMRHP8B/CMR/2014	MH933848
	EV/Human/CMRHP9/CMR/2014	MH933849
	EV/Human/CMRHP14/CMR/2014	MH933850
	EV/Human/CMRHP4/CMR/2014	MH933851
	EV/Human/CMRHP18/CMR/2014	MH933852
	EV/Human/CMRHP35A/CMR/2014	MH933853
	EV/Human/CMRHP35B/CMR/2014	MH933854
	EV/Human/CMRHP45/CMR/2014	MH933855
	EV/Human/CMRHP52A/CMR/2014	MH933856
	EV/Human/CMRHP52B/CMR/2014	MH933857
	EV/Human/CMRHP55/CMR/2014	MH933858
	EV/Human/CMRHP58/CMR/2014	MH933859
	EV/Human/CMRHP39/CMR/2014	MH933860

Supplemental Table 3B: Accession numbers of raw reads from individual pools submitted to Short Read Archive under the BioProject number PRJNA491626 and Tax ID 1861841. For ethical reasons, reads mapping to the human genome were removed.

Sample name	Accession	Sample name	Accession
HP01	SAMN10080877	HP33	SAMN10080909
HP02	SAMN10080878	HP34	SAMN10080910
HP03	SAMN10080879	HP35	SAMN10080911
HP04	SAMN10080880	HP36	SAMN10080912
HP05	SAMN10080881	HP37	SAMN10080913
HP06	SAMN10080882	HP38	SAMN10080914
HP07	SAMN10080883	HP39	SAMN10080915
HP08	SAMN10080884	HP40	SAMN10080916
HP09	SAMN10080885	HP41	SAMN10080917
HP10	SAMN10080886	HP42	SAMN10080918
HP11	SAMN10080887	HP43	SAMN10080919
HP12	SAMN10080888	HP44	SAMN10080920
HP13	SAMN10080889	HP45	SAMN10080921
HP14	SAMN10080890	HP46	SAMN10080922
HP15	SAMN10080891	HP47	SAMN10080923
HP16	SAMN10080892	HP48	SAMN10080924
HP17	SAMN10080893	HP49	SAMN10080925
HP18	SAMN10080894	HP50	SAMN10080926
HP19	SAMN10080895	HP51	SAMN10080927
HP20	SAMN10080896	HP52	SAMN10080928
HP21	SAMN10080897	HP53	SAMN10080929
HP22	SAMN10080898	HP54	SAMN10080930
HP23	SAMN10080899	HP55	SAMN10080931
HP24	SAMN10080900	HP56	SAMN10080932
HP25	SAMN10080901	HP57	SAMN10080933
HP26	SAMN10080902	HP58	SAMN10080934
HP27	SAMN10080903	HP59	SAMN10080935
HP28	SAMN10080904	HP60	SAMN10080936
HP29	SAMN10080905	HP61	SAMN10080937
HP30	SAMN10080906	HP62	SAMN10080938
HP31	SAMN10080907	HP63	SAMN10080939
HP32	SAMN10080908		
